# Supplementary material for: Patient level cost of diabetes self-management education programmes: an international evaluation
Source: BMJ Open. 2017 Jun 4;7(5):e013805. doi: 10.1136/bmjopen-2016-013805 (PMC5623445; doi:10.1136/bmjopen-2016-013805)
Supplement: Supplementary figures [file bmjopen-2016-013805supp001.pdf]

Data to Support Figures 1-5

**Programme**	Total Cost per-programme (home currency)	Total Cost per-programme (Int$)
UK 1	£1201.83	1697.50
UK 2	£883.60	1248.02
UK 3	£402.03	567.83
Taiwan	227.71 NTD	14.01
Israel 1	2532.47 ILS	633.11
Israel 2	5952.05 ILS	1484.52
Ireland 1	€923.38	1099.26
Ireland 2	€3918.31	4664.65
Ireland 3	€2682.40	3193.33
Germany 2	€451.52	573.72
Austria 1	€619.68	766.93
Austria 2	€536.28	663.71
Austria 3	€109.97	136.10

Figure 1 Data: Cost Per Programme (Salary and Overheads)

Cost Driver 1: Percentage of total personnel time used per site

**Staff (Hours) Programme**	Consultant	GP	DSN	Nurse	AHP	Dietician	Social Worker	Trained Educator	Administrator	Total time (Hours)
UK 1	-	-	6 (11%)	-	8 (14%)	4 (7%)	-	-	39 (68%)	57 (100%)
UK 2	-	-	-	-	-	-	-	15 (73%)	5.5 (27%)	20.5 (100%)
UK 3	-	-	5.5 (52%)	-	2 (19%)	3 (29%)	-	-	-	10.5 (100%)
Taiwan	-	-	-	-	-	2.4 (50%)	-	2.4 (50%)	-	4.8 (100%)
Israel 1	-	-	-	11.5 (92%)	-	-	-	-	1 (8%)	12.5 (100%)
Israel 2	-	.75 (4%)	-	.75 (4%)	4 (22%)	1.5 (8%)	9 (50%)	-	2 (12%)	18 (100%)
Ireland 1	-	-	-	-	-	-	-	14 (76%)	4.5 (24%)	18.5 (100%)
Ireland 2	-	-	56 (72%)	-	-	-	-	-	22 (28%)	78 (100%)
Ireland 3	-	-	60.6 (100%)	-	-	-	-	-	-	60.6 (100%)
Germany 2	.75 (4%)	-	-	13 (70%)	2.5 (14%)	-	-	-	2.25 (12%)	18.5 (100%)
Austria 1	-	4 (17%)	-	-	-	-	-	8 (35%)	11 (48%)	23 (100%)
Austria 2	-	4 (21%)	-	-	-	-	-	8 (42%)	7 (37%)	19 (100%)
Austria 3	-	-	-	-	-	-	-	4 (76%)	1.25 (24%)	5.25 (100%)

Figure 2 Data: Percentage of time for each personnel type per site

Cost Driver 2: Percentage of total salary cost (Int $) across personnel type and site

**Total staff cost per personnel type /prog.**	Consultant	GP	DSN	Nurse	AHP	Dietician	Social Worker	Trained Educator	Administrator	Total salary cost (Int $) per site
UK 1	-	-	264.60 (17%)	-	201.92 (13%)	100.96 (6%)	-	-	992.16 (64%)	1559.64 (100%)
UK 2	-	-	-	-	-	-	-	661.50 (73%)	121.55 (27%)	452.30 (100%)
UK 3	-	-	242.55 (66%)	-	50.48 (14%)	75.72 (20%)	-	-	-	368.75 (100%)
Taiwan	-	-	-	-	-	3.02 (50%)	-	3.02 (50%)	-	6.04 (100%)
Israel 1	-	-	-	325.60 (94%)	-	-	-	-	20.22 (6%)	345.82 (100%)
Israel 2	-	55.36 (11%)	-	21.23 (4%)	94.80 (20%)	42.47 (9%)	227.53 (47%)	-	40.45 (8%)	481.84 (100%)
Ireland 1	-	-	-	-	-	-	-	665.00 (76%)	213.75 (24%)	878.75 (100%)
Ireland 2	-	-	2897.44 (76%)	-	-	-	-	-	927.30 (24%)	3824.74 (100%)
Ireland 3	-	-	2514.90 (100%)	-	-	-	-	-	-	2514.90 (100%)
Germany 2	108.48 (26%)	-	-	264.16 (63%)	44.60 (11%)	-	-	-	-	417.24 (100%)
Austria 1	-	145.04 (18%)	-	-	-	-	-	243.60 (30%)	414.26 (52%)	802.90 (100%)
Austria 2	-	145.04 (27%)	-	-	-	-	-	243.60 (45%)	147.63 (28%)	536.27 (100%)
Austria 3	-	-	-	-	-	-	-	121.80 (82%)	26.36 (18%)	148.16 (100%)

Figure 3 Data: Weighted Average Capacity Cost Rate (Percentage of total salary cost (Int $) across personnel type and site)

Cost Driver 3: Number of Attendees at Each Programme

Programme	Number of participants
UK 1	13
UK 2	10
UK 3	9
Taiwan	1
Israel 1	10
Israel 2	15
Ireland 1	9
Ireland 2	11
Ireland 3	20
Germany 2	8
Austria 1	12
Austria 2	6
Austria 3	7*

Figure 4 Data: Number of Patients who Attend each Programme Course

*This programme runs for insulin and non-insulin users – insulin typically have 4 patient attendees and non-insulin typically have 9 patient attendees. We took the median number = 6.5 rounded to 7.

Programme	Total Cost	Price Variance from Median	Practitioner Hours	Prac Hrs Variance from Median	*Weighted Average CCR*	*Weighted Average CCR* Variance from Median	No. of Patients	No. of Patients Variance from Median
Ire 2	4664	3,898	78	55	3,825	3,022	11	-1
Ire 3	3193	2,427	61	38	2,515	1,712	20	8
UK 1	1697	931	57	34	1,560	757	13	1
Israel 2	1485	719	25	2	482	-321	15	3
UK 2	1248	482	13	-10	452	-351	10	-2
Ire 1	1099	333	19	-4	879	76	9	-3
Aus 1	766	0	23	0	803	0	12	0
Israel 1	633	-133	15	-8	346	-457	10	-2
Aus 2	633	-133	19	-4	536	-267	6	-6
Germany 2	573	-193	19	-4	417	-386	8	-4
UK 3	567	-199	12	-11	369	-434	9	-3
Aus 3	136	-630	5	-18	148	-655	7	-5
Taiwan	14	-752	5	-18	6	-797	1	-11

Median Programme = Austria 1

Figure 5 Data: Variances from the median programme (Austria 1) – variances in practitioner hours, weighted average capacity cost rate and number of patients
