# Supplementary material for: Patient level cost of diabetes self-management education programmes: an international evaluation
Source: BMJ Open. 2017 Jun 4;7(5):e013805. doi: 10.1136/bmjopen-2016-013805 (PMC5623445; doi:10.1136/bmjopen-2016-013805)
Supplement: Supplementary tables [file bmjopen-2016-013805supp002.pdf]

Table 1: Diabetes Self-Management Outcomes Framework

Program form	Outcome I. Individual DSM dispositions	Outcome II. AADE7 Behavior	Outcome III. Disease/Health
1. Program Context	4. Increase diabetes specific self-efficacy, focus of control	11. Healthy Eating	
2. Program characteristics	5. Increase diabetes knowledge	12. Being Active	18. Disease related Outcomes
3. Characteristics of program participants (Specifically health literacy)	6. Change attitudes, believes towards diabetes	13. Self-Monitoring	19. Reduced health risks
	7. Raise diabetes awareness	14. Taking Medication	20. Health related quality of life
	8. Increase perceived social support/desirability of DSM behaviors	15. Problem Solving	21. Mental comorbidity
	9. Enhance self-reflection on DSM behaviors	16. Reduced Risk Behavior	
	10. Improve DSM skills	17. Healthy coping	

Note:

The sub-categories of the DSMOF served as a basis for the selection of outcome measures for the study. Items were selected to measure diabetes self-management behaviors, health indicators, health literacy measures and program information as well as socio-demographic information. Thereby all outcome measures are self-reported, i.e. no biomarkers and no HbA1c was measured.

Table 2: Self-Reported Patient Outcomes

	N	Gen Diet	SD	Special Diet	SD	Problem Solving	SD	Reduced Risk Behaviour	SD	Health Coping	SD	Diabetes Health Literacy	SD	Fdhl	SD	CrDhl	SD
UK 1	2	0	0	1.33	.94	0.5	3.54	1.25	0.17	-1	2.83	0.07	0.2	0	0.28	0.25	0
UK2	21	0.02	.73	-0.21	1.75	0.81	2.89	0.11	0.94	1.33	3.23	-0.19	0.37	-0.3	0.48	-0.17	0.59
UK3	27	0.09	1.26	0.32	1.01	1.81	4	0.49	0.84	0.85	3.45	-0.04	0.35	-0.09	0.46	-0.01	0.69
GER2	5	0	1.87	0.07	1.44	2.6	1.5	0.89	1.11	3.14	3.58	0.13	0.11	0.11	0.3	0.26	0.5
IS1	11	0.59	1.11	-0.39	2.33	4.18	5.96	0.41	1.4	3.73	3.93	-0.03	0.27	0.07	0.59	-0.25	0.8
IS2	64	0.38	3	-0.22	2.33	0.58	5.34	0.23	1.75	1.17	4.53	0.07	0.52	0.05	0.91	0.04	0.8
IR1	13	0.73	1.59	-0.74	1.42	5	4.28	0.17	1.71	3	4.08	0.21	0.59	-0.12	0.74	0.35	0.85
IR2	5	-0.2	0.45	-0.07	1.83	4.6	5.98	0.55	1.05	3	2.74	0.2	0.45	0.36	0.26	-0.05	0.59
AUS1	6	0.5	1.1		1.48	1.5	3.62	1.38	0.92	3.5	3.08	0.07	0.25	-0.3	0.37	0.42	0.47
AUS2	2	1.5	1.12	1.83	4.95	-1.5	4.95	3	1.77	0.5	4.95	0.25	0.25	-0.2	0.42	0.5	0.28
AUS3	5	0.3	.72	0.93	3.49	1.2	3.49	0.3	1.2	4.4	3.44	-0.43	0.42	-0.28	0.46	-0.75	0.41

Figure 6 Data: Self-Reported Patient Outcomes (SD=Standard Deviation)

For Figure 6, General Diet was taken as one example of the health outcome data achieved when mapped with cost per programme. To include each health outcome would make Figure 6 too complex and the main finding that health outcomes were similar across all programmes would not be clear to the reader.

Note:

Behavioral outcomes: Behavioral self-management was assessed by selected subscales of the *Summary of Diabetes Self-care Activities* questionnaire (SDSCA). Participants thereby specified how many days in the last week they have followed a general and diabetes specific diet, exercised, checked their blood glucose level, took their prescribed medication and cared for their feet properly. In addition the reversed scores of the *Problem Areas in Diabetes* questionnaire (PAID20) and the *Appraisal of Diabetes Scale* (ADS) were used as indicators to operationalize diabetes specific problem solving and healthy coping respectively.

Covariates: In addition to the behavioral and health outcomes, socio-demographic variables and health literacy were measured as potential covariates likely to influence the effectiveness of DSM interventions. For socio-demographic variables, information on gender, age, years of education, self-perceived social status measured on a ten point scale, and ethnicity measured by own or parental migration experience was collected. Health literacy was assessed using a six item short form of the HLS-EU questionnaire and a diabetes specific health literacy (DHL) scale. The intervention dose in terms of attended sessions was also assessed, but had to be excluded from the analysis, since some respondents had difficulty in providing this information.

Table 3: Overall effectiveness of DSME programs in Aggregate (*Number of participants* = 366)

Indicator	Baseline	Follow-Up	X^2 or t	P	Cohen's d
Behavioral outcomes					
Diet (mean ± SD)	4.49 ± 2.18	4.85 ± 1.92	3.57	< .001	0.187
Diabetes specific diet (mean ± SD)	4.26 ± 1.49	4.34 ± 1.42	0.98	.163	
Exercise ≥ 1 d/wk (%)	78	84	7.22	< .001	
Blood glucose monitoring ≥ 6 d/wk (%)	28	32	3.32	.068	
Taking medication 7 d/wk (%)	88	92	5.30	.021	
Problem areas (mean ± SD)	12.63 ± 5.49	13.76 ± 5.44	5.01	< .001	0.262
Foot care (mean ± SD)	4.18 ± 1.53	4.72 ± 1.53	6.73	< .001	0.362
Appraisal of diabetes (mean ± SD)	24.77 ± 4.56	25.83 ± 4.71	5.14	< .001	0.269
Disease/health outcomes					
BMI (mean ± SD)	30.11 ± 6.60	29.89 ± 6.43	3.45	< .001	0.181
Health related quality of life (mean ± SD)	53.73 ± 19.33	57.90 ± 19.54	4.88	< .001	0.255
Affective well-being (mean ± SD)	59.24 ± 24.82	61.78 ± 23.87	2.21	.014	0.115

Figure 6 Data: Overall effectiveness of DSME programs in Aggregate (Source: Peer et al., 2016)

Note:

Disease/Health outcomes: Three disease and health outcomes were assessed: (1) the BMI was calculated to assess health risks, (2) the general health perception subscale of the *SF36* was used to quantify health related quality of life, and (3) the *WHO-5* was used to operationalize affective well-being as a reverse indicator for mental comorbidity.
