# Supplementary material for: Patient level cost of diabetes self-management education programmes: an international evaluation
Source: BMJ Open. 2017 Jun 4;7(5):e013805. doi: 10.1136/bmjopen-2016-013805 (PMC5623445; doi:10.1136/bmjopen-2016-013805)
Supplement: Author's manuscript [file bmjopen-2016-013805.draft_revisions.pdf]

BMJ Open

Patient Level Cost of Diabetes Self-Management Education Programmes: An International Evaluation

Journal:	*BMJ Open*
Manuscript ID	bmjopen-2016-013805
Article Type:	Research
Date Submitted by the Author:	12-Aug-2016

Complete List of Authors:	Doyle, Gerardine; University College Dublin, College of Business<br>O'Donnell, Shane; University College Dublin<br>Quigley, Etáin; University College Dublin<br>Cullen, Kate; University College Dublin<br>Gibney, Sarah; University College Dublin<br>Levin-Zamir, D ; University of Haifa,<br>Ganahl, Kristin; Ludwig Boltzmann Institut Health Promotion Research<br>Mueller, Gabriele; Technische Universitat Dresden<br>Muller, Ingrid; University of Southampton, Department of Psychology<br>Maindal, Helle; Aarhus University<br>Chang, Wushou; School of Public Health, Taipei Medical University; Department of Occupational Medicine, Shuang Ho and Taipei Medical University Hospital<br>Van Den Broucke, Stephan; Universite catholique de Louvain, Psychological sciences Research Institute

**Primary Subject Heading**:	Health economics
Secondary Subject Heading:	Diabetes and endocrinology, Health policy, Health services research
Keywords:	Time Driven Activity Based Costing, Health literacy, Cost, Diabetes Mellitus, Type 2, Self-Management

SCHOLARONE™ Manuscripts

Patient Level Cost of Diabetes Self-Management Education Programmes: An International Evaluation

Author and Co-author's names:
Doyle, G., O'Donnell, S., Quigley, E., Cullen, K., Gibney, S., Ganahl, G., Müller, G, Muller, I., Maindal, H.T,. Chang. P,. Van den Broucke,. S.

The name, postal address, e-mail, telephone, and fax numbers of the corresponding author.

Corresponding Author:
Professor Gerardine Doyle:
University College Dublin
UCD College of Business
Q210, Lochlainn Quinn School of Business
Belfield
Dublin 4
Ireland
gerardine.doyle@ucd.ie
Tel: +353 1 716 4717
Fax: +353 1 716 4767

The full names, institutions, city, and country of all co-authors.

**Names**	**Institutions**	**City**	**Country**
**Doyle, Gerardine**	**University College Dublin**	**Dublin**	**Ireland**
**O'Donnell, Shane**	**University College Dublin**	**Dublin**	**Ireland**
**Quigley, Etáin**	**University College Dublin**	**Dublin**	**Ireland**
**Cullen, Kate**	**University College Dublin**	**Dublin**	**Ireland**
**Gibney, Sarah**	**University College Dublin**	**Dublin**	**Ireland**
**Levin-Zamir, Diane**	**Clalit Health Services**	**Tel-Aviv**	**Israel**
**Ganahl, Kristin**	**Ludwig Boltzmann Institute**	**Vienna**	**Austria**
**Müller, Gabrielle**	**Technische Universität Dresden**	**Dresden**	**Germany**
**Muller, Ingrid**	**University of Southampton**	**Southampton**	**United Kingdom**
**Maindal, Helle Terkildsen**	**Aarhus University**	**Aarhus**	**Denmark**
**Chang, Peter**	**Taipei Medical University**	**Taipei**	**Taiwan**
**Van den Broucke, Stephan**	**Université Catholique de Louvain**	**Louvain**	**Belgium**

Key words: Diabetes Mellitus Type 2, Self-management, Cost, Time Driven Activity Based Costing, Health Literacy,

Word Count - excluding title page, references, figures and tables = 4,427.

For peer review only - http://bmjopen.bmj.com/site/about/guidelines.xhtml

Abstract

Objectives: The objective of this study was to examine the value of Time Driven Activity Based Costing (TD-ABC) in understanding the process and costs of delivering Diabetes Self-Management Education programmes (DSME) in different countries and to identify potential process improvements in the delivery of such programmes.

Setting: Outpatient settings in five European countries (Austria, Denmark, Germany, Ireland, United Kingdom) and two countries outside Europe, Taiwan and Israel.

Participants: Providers of DSME programmes across participating countries (N=15) including educators and managers.

Primary and secondary measures: Time spent by providers in the delivery of DSME and resources consumed in order to compute a cost per patient per education hour.

Results: We found significant variation of how DSME programmes are provided across and within countries. Variations in costs across different sites were caused not only by the number of educators and hours of education provided but also due to significant variations in administrative processes, curriculum and educator type. The findings highlight the value of TD-ABC in calculating a patient level cost and potential of the methodology to identify process improvements in guiding the optimal allocation of scarce resources in diabetes care, in particular for DSME that is often underfunded.

Conclusions: The results of this study will inform clinicians, managers and policy makers seeking to enhance the delivery of DSME programmes at both local and international levels. The findings highlight the benefits of adopting an TD-ABC approach to reviewing and evaluating healthcare services.

Article Summary

Strengths and limitations of this study

- Time Driven Activity Based Costing (TD-ABC) has rarely been applied to care pathways within non-acute settings and as such offers a novel perspective on understanding the costs of providing chronic disease self-management education.
- This is the first cross-national study to compare the costs of DSME across a number of countries within the EU and outside the EU to include Taiwan and Israel.
- While some self-reported health outcome data was collected as part of a wider study, clinical outcomes were not collected alongside the process and cost data, making it difficult to ascertain if value is being achieved in each of the DSME programmes included in this study.

1.0 Introduction

Type 2 diabetes mellitus is one of the major public health threats of the 21st century, currently affecting approximately 59.8 million people within Europe and 415 million worldwide [1]. Further, it has been reported that diabetes medical care accounts for a disproportionate allocation of health

For peer review only

For peer review only - http://bmjopen.bmj.com/site/about/guidelines.xhtml

service resources across the western world [1]. Developing the self-care capacity of patients is critical to determining optimal clinical, behavioural and psychosocial outcomes and therefore reducing costs [2]. Diabetes self-management education (DSME) has been shown to improve patient outcomes by reducing the onset and/or advancement of diabetes related complications; by improving quality of life; strengthening self-efficacy and personal empowerment; assisting with the development of healthy coping skills; and by reducing diabetes related distress and depression [3].

The operation and delivery of DSME varies across the international landscape. They can be either professionally led or peer led. Further, they can be group based, individually based, and increasingly IT based. In addition, DSME curricula, duration and delivery may vary substantially, both within and between countries[4]. It is well established that DSME programmes are a low cost intervention per patient and cost effective from a payer perspective. For example, a recent report published by *The Center For Health Law And Policy Innovation* (Harvard Law School) argues that cost savings can be made by public and private insurers in the United States if cost sharing were eliminated and DSME were provided free of charge to patients [5]. However, little research has explored why the costs of running such interventions vary across different health care systems and jurisdictions, or why these costs may differ. This study addresses this gap in the prior literature.

Indeed most of the economic analyses has thus far focused on establishing the cost effectiveness of DSME by comparing the cost of programmes relative to their clinical effectiveness. Such evaluations are usually based on economic modelling, carried out alongside randomised control trials and the findings typically suggest that DSME interventions are cost effective relative to usual care [6-12]. Despite this, a recent report published by the *Health Information and Quality Authority* (HIQA) [13] in Ireland highlights the large degree heterogeneity in the methodological approaches used in such economic evaluations. This, in turn, makes results difficult to compare in any meaningful way. In addition, these approaches tend to focus solely on overall cost of running the programmes and neglect to explore potential mechanisms through which DSME programmes could be made more efficient whilst also maintaining high standards of effectiveness. Furthermore, the majority of studies are based on interventions within a US population, and as such may not be generalizable across differing health care, social and cultural contexts.

This study seeks to address these existing gaps in the literature through an economic evaluation of DSME delivery across a number of EU and non-EU countries, namely Austria, Denmark, Germany, Ireland, Israel, Taiwan and the UK. The selection of these countries was based on the access of the

For peer review only - http://bmjopen.bmj.com/site/about/guidelines.xhtml

Diabetes Literacy Consortium[1] to local knowledge and networks required to carry out the necessary fieldwork. These countries also represent a diversity of contrasting approaches to the delivery of DSME tailored to each country [4]. The findings are part of a wider study conducted by the Diabetes Literacy Consortium, the overall purpose of which was to examine the (cost)-effectiveness of diabetes education across Europe, Israel, Taiwan and the US[2]. This study specifically addresses the following research questions: i.) What is the cost of delivering DSME programmes? ii.) Is TD-ABC a suitable methodology for computing a patient level cost i.e. a cost per patient per education hour? iii.) Can TD-ABC aid in identifying process improvements in the delivery of DMSE programmes?

2.0 Method

A Time Driven Activity Based (TD-ABC) costing method was used to map the process of programme delivery and to derive patient level costs[14 15]. TD-ABC has been developed as a viable costing method with the healthcare sector by Kaplan and Porter [16 17] enabling detailed patient level costs to be computed alongside the identification of possible process improvements resulting in potential cost savings. TD-ABC is particularly compatible with type 2 diabetes care as the model can be applied to diverse care pathways, particularly chronic disease management. Adopting a TD-ABC approach in this study therefore gave increased visibility into areas of DSME delivery where process improvements and cost savings could be made, while still maintaining a high quality of patient education. Examples of the application of TD-ABC have been mostly confined to acute clinical settings[17-19] This study seeks to add to the body of knowledge on the costs of care within outpatient environments through identifying the patient level cost of a variety of DSME programmes both *cross*-nationally and *Intra*nationally[20] . A primary objective was to provide a robust costing framework from which future studies could include clinical and quality of life outcomes to determine the economic value added to diabetes care through the use of DSME.

The TD-ABC method involves seven steps [16] 1) select the medical condition and/or patient population to be examined; 2) define the care value chain; 3) develop process maps of each activity in patient care delivery; identify the resources involved and any supplies used for the patient at each process step; 4) obtain time estimates for each process step; 5) estimate the cost of supplying each patient care resource; 6) estimate the practical capacity of each resource provided and calculate the capacity cost rate; 7) compute the total costs over each patient's cycle of care. By constructing a

[1] The Diabetes Literacy Consortium represents a group of countries funded by the European Commission under the Seventh Framework research programme (Grant Agreement Number: 306186).
[2] http://www.diabetesliteracy.eu

For peer review only - http://bmjopen.bmj.com/site/about/guidelines.xhtml

sequential activity and process step map and care value chain the researcher can analyse the maps/care pathway for duplication. These areas can then be explored further to establish if changes in the pathway would add value by maintaining/increasing the level of care to the patient whilst decreasing the economic cost to the overall healthcare system in terms of providing DSME programmes.

Each international study team identified the care value pathway in their country and collected the activity/time data related to the care value pathway through qualitative semi-structured interviews of healthcare providers from each education programme (N=15). These included both educators and managers. This information was then entered into an aggregated, de-identified database for analysis. All study teams then collected resource and financial data, utilising a standardised costing worksheet related to the activities, which were then incorporated into the aggregated database for analysis. This methodology was applied to each education programme across each country included in the study. The topic guide was developed in the English language and was then subsequently translated into the local language by the local research teams in each of the participating countries.

All activities associated with the DSME pathway were entered into an aggregate Excel database. All activity and time data was collected via the survey instrument, and cost estimates were assigned to these activity variables using financial data provided by the local provider organizations.

DSME programme costs per patient were derived specifically from the cost of performing each activity in the delivery of the programme. All cost data was entered into activity spreadsheets and therefore the data collected did not contain any information relating to identifiable individual service providers. In the resulting database, all cost information was linked to activities and not to individuals. All activity and cost information is reported per DSME programme.

2.1 Study Sample

To be selected for inclusion, programmes had to: (1) target type 2 diabetes patients; (2) be conducted among the general patient population rather than tailored to the needs of a specific age cohort, needs or gender group; (3) include (but not be limited to) newly diagnosed patients; (4) be stand-alone programmes rather than an add-on to another programme or part of a wider curriculum with (multiple) parallel programs; (5) admit new patients during the time of the baseline data collection. The study sample size was driven by the number of programmes involved in the delivery of the specific DSME programmes in each country. Table 1 outlines the number of programmes per

For peer review only - http://bmjopen.bmj.com/site/about/guidelines.xhtml

country eligible for inclusion in the study. Costs were collected for the duration of each programme, which ranged in duration from one day to those spanning a 12-month timeframe.

Table 1: Study Sample

COUNTRY	SAMPLE SIZE
AUSTRIA	3
DENMARK	1
GERMANY	3
IRELAND	3
ISRAEL	2
TAIWAN	1
UK	3

2.2 Analytic approach

The Time-Driven Activity-Based Costing (TD-ABC) model was utilised to derive a cost per patient per hour for each education programme studied. This cost figure was then used to compare the programme cost per patient across the various programmes.

Two concepts and measures were drawn upon to develop the TD-ABC model [16],the unit cost of supplying capacity and the time it takes to undertake an activity. First, the model was used to calculate the cost of all the resources supplied to each programme. This included personnel, supervision and overheads rent, equipment and software and insurance. The total cost was then divided by the actual capacity in order to calculate the cost rate. Second, the capacity cost rate was used to assign the programme costs to objects by estimating demand on the resource. Two variables were estimated: the capacity cost rate for the programme and the capacity use by each patient. The capacity cost rate was calculated by:

$$\text{Capacity Cost Rate} = \frac{\text{Cost of Capacity Supplied}}{\text{Practical Capacity of Resource Supplied}}$$

Practical capacity was used as the denominator in the capacity cost rate equation. Estimating the practical capacity required two time estimates which were gathered from Human Resources and other administrative records: the total number of days that each employee actually worked each year; the total number of hours per day that the employee was available for work. Practical capacity was calculated as 80% of this working time [16]. Therefore 20% was attributed to breaks, training and

For peer review only - http://bmjopen.bmj.com/site/about/guidelines.xhtml

annual leave. This was applied to all countries to ensure consistency and comparability of the computed costs.

In order to calculate the total cost of each DSME programme per patient, the capacity cost rates (including associated support costs) for each resource used was multiplied by the amount of time attributed to each patient. This calculation was based on the number of patients enrolled at the outset of the programme. The total cost of each programme per patient was the sum of all the costs across all the processes within the DSME programme. The costs were collected in the local currency and then expressed in international dollar to ensure comparability of the cost per patient per hour.

3.0 Results

Table 2 presents the results for each programme for each country included in the study. It outlines the anonymised programme name, type of professional educator, the number of sessions per programme, the hours of education per programme, the hours of administrative work/preparation time per programme, number of patients per programme, cost per patient per programme and cost per patient for each education hour, and finally the cost per patient per education hour is expressed in International dollar.

For peer review only

Table 2: Results Table

Country	Programme	Educator	No. sessions per programme	Hours of education per programme	Hours of admin/prep per programme	No. patients per programme.	Cost per patient per programme	Cost per patient per hour	Cost per patient per hour (Int. $*)[34]
**AUSTRIA**	1	Diabetes educator, Dietician, Physician	5	20	9	12	€41.73	€2.03	**$2.40**
	2	Dietician, Physician	4	10	7	6	€72.22	€7.22	**$9.05**
	3	Diabetes Educator	7	4.4	1.25	4	€6.23	€1.56	**$1.93**
**DENMARK**	1	Peer led educator x 2	12	30	16.6	11	€34.47	€1.14	**$.55**
**GERMANY**	1	Nutritionist	5	3	.5	1	€70.31	€23.44	**$29.78**
	2	Diabetes nurse	5	11.25	2.25	8	€44.42	€3.95	**$5.02**
	3 (opp. cost)	Diabetes patient trained to provide peer-led education	10	15	60	20	€8.76	€0.58	**$0.74**
**IRELAND**	1	Specially trained health professional	1 or 2	7	4.5	9	€86.37	€12.34	**$14.69**
	2 (site 1)	Trained dietician	8	23	55	11	€299.22	€13.00	**$15.48**

peer review only

For peer review only - http://bmjopen.bmj.com/site/about/guidelines.xhtml

	2 (site 2)	Trained dietician	8	22	38.6	20	€112.83	€5.13	**$6.11**
**ISRAEL**	1	Specially trained nurse	4	7	8	10	416.62 NIS	58.87 NIS	**$14.68**
	2	Social worker, GP, diabetes nurse, physiotherapist/ exercise specialist, dietician	6	9	10.75	15	542.43 NIS	60.38 NIS	**$15.28**
**TAIWAN**	1	Diabetes educator	5	1.8	n/a	1	110.33 NTD	61.00 NTD	**$3.75**
**UK**	1	Diabetes specialist nurse, dietician, physiotherapist, podiatrist	5	10	39	13	£78.62	£7.86	**$11.10**
	2	Specially trained health care professionals	1 or 2	7.5	5.5	10	£74.59	£9.93	**$14.03**
	3	Diabetes specialist nurse, dietician, podiatrist	2	6	6.7	9	£37.71	£6.28	**$8.87**

Table 2: Demonstrates the costs of delivering DSME, expressed in international dollar. An international dollar is a hypothetical currency that is used as a means of translating and comparing costs from one country to the other using a common reference point. International dollars are calculated by dividing the DSME cost in a country's home currency by its relevant Purchasing Power Parity conversion factor. The use of the PPP technique minimizes misleading cost comparisons between countries with the use of exchange rates alone. PPP conversion factors are constructed by comparing the national prices of a large basket of goods and services and these rates are then used to translate different currencies into a common currency. The PPP conversion factors used in this study were sourced from the OECD (http://stats.oecd.org/Index.aspx?DataSetCode=PPPGDP) and based on 2014 exchange rates. The PPP conversion factors used were as follows: UK 0.708; Israel 4.01; Taiwan 16.25; Ireland 0.84; and Germany 0.787; Denmark 7.48 and Austria 0.808 and were sourced from http://stats.oecd.org/Index.aspx?DataSetCode=PPPGDP. Opportunity cost is utilized when the provider of the service/care is not a paid employee, such as in the case of peer led programmes based in Germany and Denmark. This form of cost is considered a measure of economic sacrifice related to the time provided. Calculations are made based upon 25% of the average industrial wage of the related country based on an assumption that leisure time is being sacrificed [21] All those included in the study reported sacrificing leisure time.

For peer review only

For peer review only - http://bmjopen.bmj.com/site/about/guidelines.xhtml

Austria

Three programmes from Austria were included in the study. All were group led sessions and had varying practitioners providing the education (see table 2 above). Programme 1 cost €41.73 per patient which equates to €2.03 per patient per hour. When converted into international dollar this programme cost $2.40 per patient per hour. Programme 2 cost is €72, equating to €7.22 per patient per hour, converted to the international dollar, this programme cost $9.05 per patient per hour. Programme 3 cost is €109, equating to €1.56 per patient per hour, when converted into the international dollar costs $1.93 per hour. This data illustrates the significant variation in cost that exists within jurisdictions. This is due to significant variations between the programmes in the number of hours of education, the number of patients participating in these programmes and the number of administration hours.

Denmark

Denmark operates both professional-led (from hospital settings and local health centres) and peer-led diabetes self-management education programmes. One peer led programme was included in this study from Denmark (data was not available for the professionally led programmes). This programme cost €34.47 per patient per programme which equates to €1.14 per patient per hour. When converted into international dollar this programme cost $0.15 per patient per hour. This finding highlights the low cost of peer-led programmes per patient per hour in Denmark.

Germany

Germany operates both professional and peer-led diabetes self-management education programmes. This study included both programmes due to the high level of peer-led programmes offered within the German health care system. Programme 1 (professional) cost €70.32 per patient which equates to €23.44 per patient per hour. When converted into international dollar this programme cost $29.78 per patient per hour; programme 2 (professional) cost €44.42 per patient which equates to €3.95 per patient per hour. When converted into international dollar this programme cost $5.02 per patient per hour; while programme 3 (peer-led) cost €8.76 per patient which equates to €0.58 per patient per hour. When converted into international dollar this programme cost $0.72 per patient per hour. Here too we observe a significant variation in cost across programmes within the same country. In particular the low cost of peer-led education as was found in Denmark.

For peer review only - http://bmjopen.bmj.com/site/about/guidelines.xhtml

Ireland

Two programmes from Ireland were included in the study and both programmes were group based. Programme 1 cost €86.37 per patient which equates to €12.34 per patient per hour. When converted into international dollar this programme cost $14.69 per patient per hour. Programme 2 (site 1) cost €299.22 per patient per programme which equates to €13.00 per patient per hour. When converted into international dollar this programme cost $15.48 per patient per hour; while programme 2 (offered in site 2) cost €112.83 per patient per programme which equates to €5.13 per patient per hour. When converted into international dollar this programme cost $6.11 per patient per hour. Significant cost variation within a country is again evident. However what is most significant is the different costs within a single programme when delivered across different sites. For example Programme 2 had different administrative hours (55 versus 39 hours), different personnel performing the administrative tasks (a clinical nurse specialist versus an administrator) and different numbers of patient per programme.

Israel

Two programmes from Israel satisfied the inclusion criteria for this study and both programmes provide group based education. Programme 1 cost 416.62 NIS per patient which equates to 58.87 NIS per patient per hour. When converted into international dollar this programme cost $14.68 per patient per hour and programme 2 cost 523.43 NIS per patient, equating to 60.38 NIS per patient per hour. When converted into international dollar this programme cost $15.28 per patient per hour. The costs for both programmes are almost identical despite the different mix of educators, the variation in terms of the number of sessions provided, and the greater number of hours of education and administrative hours. This can be explained by the greater number of patients being included in Programme 2 (10 in programme 1 versus 15 in Programme 2) thus leading to only a small cost differential between the programmes despite the variations in how the programmes were run.

Taiwan

One programme from Taiwan and was included in the study. This programme is provided as an individualised or group education programme. One on one Individual programmes are the norm. Group sessions are performed only when there are insufficient numbers of staff to perform individualised education. This programme cost 110.33 DT per patient which equates to 61.00 DT per patient per hour. When converted into international dollar this programme cost $3.75 per patient per hour. The low cost reflects the low number of contact hours for patients, being only 1.8 hours per programme.

For peer review only - http://bmjopen.bmj.com/site/about/guidelines.xhtml

United Kingdom

A total of three UK based programmes satisfied the inclusion criteria for this study. All programmes provide group sessions. Programme 1 cost £78.62 per patient which equates to £7.86 per patient per hour. When converted into international dollar this programme cost $11.10 per patient per hour; Programme 2 cost £74.59 per patient equating to £9.93 per patient per hour. When converted into international dollar this programme cost $14.03 per patient per hour; and Programme 3 cost £37.71 per patient per programme which equates to £6.28 per patient per hour. When converted into international dollar this programme cost $8.87 per patient per hour. Once again significant cost variation exists within this country, reflecting significantly high administrative hours in Programme 1. Within Programme 2 we note specially trained healthcare professionals providing the education for this programme. This results in a comparatively high cost per patient per programme despite a lower number of contact hours (7.5 versus 10 hours).

4.0 Discussion

Alongside the specific country data Table 2 demonstrates that there is a significant variation of how DSME is provided across countries – the site/institution where the programme is provided, by whom it is provided, the number of sessions per programme, the number of education hours provided, and the curriculum. Further, it highlights that costs differ as a result of these variances in approaches. In particular findings suggest that a number of programmes have extremely high administrative costs associated with the delivery of DSME programmes, this is particularly the case in the UK and Ireland. In other countries, the administrative costs attached to the programmes appear low. This finding is similar to that of Munoz et al. who used TD-ABC in a cost-effectiveness analysis of a red blood cell salvage post total-knee arthroscopy in the US, Switzerland and Austria and highlighted the need for local cost estimations in place of global cost estimates in future replications in cost-effectiveness analysis for this particular procedure[22].

Whilst it is accepted that administration time/costs related to delivering education, particularly when provided through group sessions, may be significant, future studies could examine what processes and protocols could be put in place in order reduce the number of hours of personnel time spent on administration. Indeed research by Storfjell et al., has shown that the application of TD-ABC in the context of nursing care can facilitate the identification and elimination of non-value added time (NVA) and related the increase in time spent on psychosocial intervention, support and patient education[23].

For peer review only - http://bmjopen.bmj.com/site/about/guidelines.xhtml

Findings also point to the significant differences between the various personnel who deliver the education programmes. In this sense research is needed to explore the most appropriate level of expertise required to deliver the programme for optimal patient health outcomes. For example, instead of having a consultant or a Clinical Nurse Specialist delivering the education programmes it may be more appropriate to have well trained experienced nurses or the equivalent performing this role. Furthermore, little attention has been paid to peer-led programmes in terms of their effectiveness. Table 2 illustrates that peer-led programmes cost significantly less than professionally led programmes. Managers may also want to consider whether resources are being deployed effectively within education programmes and whether the expertise would provide better value (in terms of patients' outcomes) elsewhere in the healthcare system. For example one pilot study conducted by Kaplan et al. at The University of Texas Cancer Centre revealed that the matching of clinical skills to the processes led in a 16% reduction in process time, a 12% decrease in costs for technical staff, and a 67% reduction in costs for professional staff[16]. However, clinical outcomes data will first be required in order to examine whether the educators level of expertise influences DSME health outcomes[16].

This supports and further highlights what was discussed at the outset of this paper, namely, that the costing and provision of DSME is at an early stage of development with limited empirical knowledge attached to the various strands of its delivery. Thus this study has gone some way to remedying this problem whereby it has outlined a bottom up/patient level cost, and therefore more accurate cost than heretofore, of providing the various education programmes. Thus, it has provided a first layer of information, which in the future will be required to establish whether this model of care/intervention can add value to the health care system once effectiveness outcomes have been determined for each programme, However, there is a long way to go, whereby clinical and Quality of Life outcomes are required to measure the effectiveness of these programmes before a thorough understanding of their added value can be estimated. In this respect it is suggested that future research should explore these areas with a view to using the data from this study to develop a better understanding of the added value derived from providing DSME interventions.

5.0 Limitations

The TD-ABC method is a relatively new method in terms of healthcare costing and to the best of the authors' knowledge has yet to been applied to investigate the costs of a health education

For peer review only - http://bmjopen.bmj.com/site/about/guidelines.xhtml

intervention. As a result there were limited guidelines surrounding the collection of activity and process step data in non-acute settings and thus it was necessary for the research team to develop novel data collection tools to meet the requirements of the model. Whilst the tools generated data required to answer the research question, there were many lessons learned from this phase of the Diabetes Literacy project. Some participants reported difficulties in filling out the survey as a result of a lack of familiarity with the terminology surrounding activities and process steps. This resulted from a lack of understanding related to the granular level of information that was requested by the project researchers. Therefore some elements of the detailed administrative process steps were lost. For example, surveys were completed in a manner which reflected the activities and process steps related to the education activity but less attention was paid by some respondents to providing the same level of detail about the administrative and preparation activities and process steps. This detailed information would have provided greater insight into the reasons why administrative costs were found to be so high in some countries while not in others. For example, in Ireland the same programme, reviewed at two different sites, had significant variation in terms of the administrative/preparation time attached to each session provided (55 hours versus 39 hours). Moreover, in the UK one programme had almost three times the administrative hours attached to each session when compared to educational contact hours (10 hours educational contact time versus 39 hours administrative/preparation time). Yet, in Austria educational contact time allocated per session was just over double the time attributed to administration/preparation time (20 hours educational contact versus 9 hours administration/preparation).

In addition, some of the local research teams also experienced difficulties in collecting the required financial data. For example, in Belgium, the staff involved in the delivery of DSME programmes taking part in this study were unwilling to share salary information at the level of granularity that was required to compute a per patient cost. For this reason, the Belgian data had to be excluded from this particular study.

The study is also limited by a lack of available clinical outcome data from each of the education programmes to allow for comparison with cost. While evaluative data was collected in each country as part of the FP7 study, it was almost exclusively self-reported in nature, making it difficult to substantiate if value was being achieved by these DSME programmes. As Kaplan and Porter point out[16], value can only be determined when there is visibility into both costs and clinical outcomes.

For peer review only - http://bmjopen.bmj.com/site/about/guidelines.xhtml

6.0 Conclusion

This paper has demonstrated the variances in the cost of delivering different types of diabetes education programmes, both within and across countries in the EU, as well as Israel and Taiwan. Developing cost effective lifestyle interventions to improve the health literacy and quality of life for people with diabetes may be an important step in preventing the onset of complications associated with type 2 diabetes. The imperative to do so from an economic perspective cannot be underestimated when consideration is given to the implications for healthcare systems associated with the treatment of diabetes related morbidities such as active foot disease, chronic kidney disease, retinopathy and myocardial infarction[24].

This study offers the first application of a TD-ABC approach to evaluate the cost of delivering DSME programmes and as a means of comparing the costs of running a healthcare intervention cross-nationally. It contributes to the extant literature by highlighting and describing the vast combinations and permutations of DSME curricula, hours of education, educators, and numbers of attendees and how these variations lead to substantial cost differences. In the process, we identified how there could be potentially unnecessary process steps that, if eliminated, could lead to cost savings in the delivery of DSME programmes, including vast differences in administration time, and exploring different types of personnel delivering the programmes. To what extent value can be improved in these areas can only be determined through future studies.

While it is already established that diabetes education is a low cost intervention and is cost-effective, given the sheer numbers of education programmes that need to be made available to meet the demands resulting from increasing levels of diabetes worldwide, even small process improvements could lead to overall cost savings for healthcare providers. Future studies focusing on the cost-effectiveness of healthcare interventions may consider adopting TD-ABC principles as a means of identifying efficiencies in other chronic disease education programmes.

The study has highlighted the strengths of TD-ABC as a method of bottom up costing in outpatient care and recommends utilising this method in future studies so as to allow for a comprehensive literature to develop in the area, enabling comparative studies to be performed. By developing such literature a comprehensive understanding of the cost of patient education programmes can be developed and compared cross nationally and across time. Healthcare practitioners and educators who wish to convince policy makers and health insurers to reimburse the cost of DSME delivery can adopt a TD-ABC approach in order to demonstrate that such programmes are run efficiently and

For peer review only - http://bmjopen.bmj.com/site/about/guidelines.xhtml

effectively especially when combined with measures of consequent health outcomes to represent value for money.

Acknowledgements

This article is part of the Diabetes Literacy project supported by grant FP7-Health-2012-Innovation-1/306186 of the European Commission. The authors would like to thank the partners of the Diabetes Literacy Consortium for contributing to data collection with participants and providers of DSME programs in their respective countries.

The Diabetes Literacy Consortium consists of the following members:

Université Catholique de Louvain, Belgium: Stephan Van den Broucke, Gerard Van der Zanden, Marie Housiaux, Louise Schinckus.

Technische Universität Dresden, Medical Faculty, Germany: Peter Schwarz, Gabriele Mueller, Henna Riemenschneider, Sarama Saha.

University College Dublin, Ireland: Gerardine Doyle, Shane O'Donnell, Etain Quigley, Kate Cullen, Sarah Gibney

Ludwig Boltzmann Institute for Health Promotion Research, Austria: Jürgen Pelikan, Florian Röthling, Kristin Ganahl, Sandra Peer.

Maastricht University, Department of International Health, The Netherlands: Helmut Brand, Kristine Sörensen, Timo Clemens, Marjo Campmans

University of Southampton, UK: Lucy Yardley, Ali Rowsell, Ingrid Muller, Victoria Hayter

Clalit Health Services, Israel: Diane Levin-Zamir, Ziv Har-Gil.

University of California at San Francisco, USA: Dean Schillinger, Courtney Lyles, Lina Tieu.

Taipei Medical University, Taiwan: Peter Chang, Candy Kuo, Alice Lin, Duong Van Tuyen, Becky Sun.

Aarhus University, Denmark: Helle Terkildsen Maindal, Jill Rowlands, Ulrik Martensen.

We also thank all the medical and educational services from the eight participating countries who contributed to the data collection.

For peer review only - http://bmjopen.bmj.com/site/about/guidelines.xhtml

Contributorship statement GD initially proposed the study. GD and SG specified the methodology. KC carried out the cost analysis. All authors contributed to the protocol design, data collection and analysis plan. EQ and SO'D wrote the initial manuscript, and all authors contributed to improving the manuscript. All authors approved the final manuscript.

Competing interests None declared.

Ethics Approval All methods were approved by the SVUH group Research and Ethics Committee, by the Research Ethics Committee of the Office of Research Ethics, University College Dublin and by the relevant local ethics committees in each jurisdiction and each study site where the study was carried out.

Funding This study is part of the Diabetes Literacy project supported by grant FP7-Health-2012-Innovation 1/306186 of the European Commission.

Data sharing statement The Excel spreadsheets showing how the individual activity costs were aggregated are available should they be requested.

For peer review only

References

1. International Diabetes Federation. IDF Diabetes Atlas. 7 ed ed. Brussels: International Diabetes Federation, 2015.
2. Peyrot M, Burns KK, Davies M, et al. Diabetes Attitudes Wishes and Needs 2 (DAWN2): A multinational, multi-stakeholder study of psychosocial issues in diabetes and person-centred diabetes care. Diabetes Research and Clinical Practice 2013;**99**(2):174-84.
3. Powers MA, Bardsley J, Cypress M, et al. Diabetes Self-management Education and Support in Type 2 Diabetes A Joint Position Statement of the American Diabetes Association, the American Association of Diabetes Educators, and the Academy of Nutrition and Dietetics. The Diabetes Educator 2015;**41**(4):417-30.
4. Saha S, Müller G., Riemenschneider H, et al. Compendium of Diabetes Self-Management Education Programs in the European Union Member States, Israel, Taiwan and the USA. Version 1.1
Dresden, 2015.
5. Garfield K, Downer S, Rosenberg A, et al. Reconsidering Cost Sharing for DSME: Recommendation for Policy Reform. Massachusetts: Harvard, 2015.
6. Gillespie P, O'Shea E, Paul G, et al. Cost effectiveness of peer support for type 2 diabetes. International journal of technology assessment in health care 2012;**28**(01):3-11.
7. Brown 3rd H, Wilson KJ, Pagán JA, et al. Cost-effectiveness analysis of a community health worker intervention for low-income Hispanic adults with diabetes. Prev Chronic Dis 2012;**9**:E140.
8. Brownson CA, Hoerger TJ, Fisher EB, et al. Cost-effectiveness of Diabetes Self-management Programs in Community Primary Care Settings. The Diabetes Educator 2009;**35**(5):761-69.
9. Kuo S, Bryce CL, Zgibor JC, et al. Cost-Effectiveness of Implementing the Chronic Care Model for Diabetes Care in a Military Population. Journal of Diabetes Science and Technology 2011;**5**(3):501-13.
10. Jacobs-van der Bruggen MA, van Baal PH, Hoogenveen RT, et al. Cost-effectiveness of lifestyle modification in diabetic patients. Diabetes Care 2009;**32**(8):1453-58.

For peer review only - http://bmjopen.bmj.com/site/about/guidelines.xhtml

11. Dall TM, Roary M, Yang W, et al. Health care use and costs for participants in a diabetes disease management program, United States, 2007–2008. Prev Chronic Dis 2011;**8**(3):A53.
12. Molsted S, Tribler J, Poulsen PB, et al. The effects and costs of a group-based education programme for self-management of patients with Type 2 diabetes. A community-based study. Health Education Research 2012;**27**(5):804-13.
13. Health Information and Quality Authority. Health technology assessment of chronic disease self-management support interventions. Dublin: HIQA, 2015.
14. Kaplan RS, Anderson SR. Time-driven activity-based costing. Available at SSRN 485443 2003.
15. Kaplan RS, Anderson SR. The innovation of time-driven activity-based costing. Journal of cost management 2007;**21**(2):5-15.
16. Kaplan RS, Porter ME. How to solve the cost crisis in health care. Harvard Business Review 2011;**89**(9):46-52.
17. Erhun F, Mistry B, Platchek T, et al. Time-driven activity-based costing of multivessel coronary artery bypass grafting across national boundaries to identify improvement opportunities: study protocol. BMJ open 2015;**5**(8):e008765.
18. Akhavan S, Ward L, Bozic KJ. Time-driven Activity-based Costing More Accurately Reflects Costs in Arthroplasty Surgery. Clinical Orthopaedics and Related Research® 2016;**474**(1):8-15.
19. Kaplan AL, Agarwal N, Setlur NP, et al. Measuring the cost of care in benign prostatic hyperplasia using time-driven activity-based costing (TDABC). Healthcare 2015;**3**(1):43-48.
20. Demeere N, Stouthuysen K, Roodhooft F. Time-driven activity-based costing in an outpatient clinic environment: Development, relevance and managerial impact. Health Policy 2009;**92**(2–3):296-304.
21. Connolly S, Gillespie P, O'Shea E, et al. Estimating the economic and social costs of dementia in Ireland. Dementia 2014;**13**(1):5-22.
22. Muñoz M, Campos A, Martín-Montañez E, et al. Could cost-effectiveness of postoperative shed blood salvage after elective knee arthroplasty be improved? Transfusion 2013;**53**(6):1372-73.
23. Storfjell JL, Ohlson S, Omoike O, et al. Non-Value-Added Time: The Million Dollar Nursing Opportunity. Journal of Nursing Administration 2009;**39**(1):38-45.
24. Seuring T, Archangelidi O, Suhrcke M. The Economic Costs of Type 2 Diabetes: A Global Systematic Review. PharmacoEconomics 2015;**33**(8):811-31.

For peer review only

For peer review only - http://bmjopen.bmj.com/site/about/guidelines.xhtml

CHEERS Checklist

Items to include when reporting economic evaluations of health interventions

The **ISPOR CHEERS Task Force Report**, *Consolidated Health Economic Evaluation Reporting Standards (CHEERS)—Explanation and Elaboration: A Report of the ISPOR Health Economic Evaluations Publication Guidelines Good Reporting Practices Task Force*, provides examples and further discussion of the 24-item CHEERS Checklist and the CHEERS Statement. It may be accessed via the *Value in Health* or via the ISPOR Health Economic Evaluation Publication Guidelines – CHEERS: Good Reporting Practices webpage: http://www.ispor.org/TaskForces/EconomicPubGuidelines.asp

Section/item	Item No	Recommendation	Reported on page No/ line No
**Title and abstract**			
Title	1	Identify the study as an economic evaluation or use more specific terms such as "cost-effectiveness analysis", and describe the interventions compared.	p.3 121
Abstract	2	Provide a structured summary of objectives, perspective, setting, methods (including study design and inputs), results (including base case and uncertainty analyses), and conclusions.	p.1 49-73
**Introduction**			
Background and objectives	3	Provide an explicit statement of the broader context for the study. Present the study question and its relevance for health policy or practice decisions.	p.3 104
**Methods**			
Target population and subgroups	4	Describe characteristics of the base case population and subgroups analysed, including why they were chosen.	p.4 163-166
Setting and location	5	State relevant aspects of the system(s) in which the decision(s) need(s) to be made.	p.4 145
Study perspective	6	Describe the perspective of the study and relate this to the costs being evaluated.	p.4 138
Comparators	7	Describe the interventions or strategies being compared and state why they were chosen.	p.3 123-125
Time horizon	8	State the time horizon(s) over which costs and consequences are being evaluated and say why appropriate.	p.5 192/193
Discount rate	9	Report the choice of discount rate(s) used for costs and outcomes and say why appropriate.	N/A
Choice of health outcomes	10	Describe what outcomes were used as the measure(s) of benefit in the evaluation and their relevance for the type of analysis performed.	p.4 137
Measurement of effectiveness	11a	*Single study-based estimates:* Describe fully the design features of the single effectiveness study and why the single study was a sufficient source of clinical effectiveness data.	p.14 413

For peer review only

	11b	*Synthesis-based estimates:* Describe fully the methods used for identification of included studies and synthesis of clinical effectiveness data.	N/A
Measurement and valuation of preference based outcomes	12	If applicable, describe the population and methods used to elicit preferences for outcomes.	N/A
Estimating resources and costs	13a	*Single study-based economic evaluation:* Describe approaches used to estimate resource use associated with the alternative interventions. Describe primary or secondary research methods for valuing each resource item in terms of its unit cost. Describe any adjustments made to approximate to opportunity costs.	p.4 151-156
	13b	*Model-based economic evaluation:* Describe approaches and data sources used to estimate resource use associated with model health states. Describe primary or secondary research methods for valuing each resource item in terms of its unit cost. Describe any adjustments made to approximate to opportunity costs.	N/A
Currency, price date, and conversion	14	Report the dates of the estimated resource quantities and unit costs. Describe methods for adjusting estimated unit costs to the year of reported costs if necessary. Describe methods for converting costs into a common currency base and the exchange rate.	p. 9 240-247
Choice of model	15	Describe and give reasons for the specific type of decision-analytical model used. Providing a figure to show model structure is strongly recommended.	N/A
Assumptions	16	Describe all structural or other assumptions underpinning the decision-analytical model.	N/A
Analytical methods	17	Describe all analytical methods supporting the evaluation. This could include methods for dealing with skewed, missing, or censored data; extrapolation methods; methods for pooling data; approaches to validate or make adjustments (such as half cycle corrections) to a model; and methods for handling population heterogeneity and uncertainty.	N/A
**Results**			
Study parameters	18	Report the values, ranges, references, and, if used, probability distributions for all parameters. Report reasons or sources for distributions used to represent uncertainty where appropriate. Providing a table to show the input values is strongly recommended.	N/A
Incremental costs and outcomes	19	For each intervention, report mean values for the main categories of estimated costs and outcomes of interest, as well as mean differences between the comparator groups. If applicable, report incremental cost-effectiveness ratios.	N/A
Characterising uncertainty	20a	*Single study-based economic evaluation:* Describe the effects of sampling uncertainty for the estimated incremental cost and incremental effectiveness parameters, together with the impact	N/A

For peer review only

		of methodological assumptions (such as discount rate, study perspective).	N/A
	20b	*Model-based economic evaluation:* Describe the effects on the results of uncertainty for all input parameters, and uncertainty related to the structure of the model and assumptions.	N/A
Characterising heterogeneity	21	If applicable, report differences in costs, outcomes, or cost-effectiveness that can be explained by variations between subgroups of patients with different baseline characteristics or other observed variability in effects that are not reducible by more information.	p.13 352-358
**Discussion**			
Study findings, limitations, generalisability, and current knowledge	22	Summarise key study findings and describe how they support the conclusions reached. Discuss limitations and the generalisability of the findings and how the findings fit with current knowledge.	p.12-14 331-417
**Other**			
Source of funding	23	Describe how the study was funded and the role of the funder in the identification, design, conduct, and reporting of the analysis. Describe other non-monetary sources of support.	p.16 460-463
Conflicts of interest	24	Describe any potential for conflict of interest of study contributors in accordance with journal policy. In the absence of a journal policy, we recommend authors comply with International Committee of Medical Journal Editors recommendations.	p. 16 492

For consistency, the CHEERS Statement checklist format is based on the format of the CONSORT statement checklist

The **ISPOR CHEERS Task Force Report** provides examples and further discussion of the 24-item CHEERS Checklist and the CHEERS Statement. It may be accessed via the *Value in Health* link or via the ISPOR Health Economic Evaluation Publication Guidelines – CHEERS: Good Reporting Practices webpage: http://www.ispor.org/TaskForces/EconomicPubGuidelines.asp

The citation for the CHEERS Task Force Report is:

Husereau D, Drummond M, Petrou S, et al. Consolidated health economic evaluation reporting standards (CHEERS)—Explanation and elaboration: A report of the ISPOR health economic evaluations publication guidelines good reporting practices task force. Value Health 2013;16:231-50.

For peer review only

BMJ Open

Patient Level Cost of Diabetes Self-Management Education Programmes: An International Evaluation

Journal:	*BMJ Open*
Manuscript ID	bmjopen-2016-013805.R1
Article Type:	Research
Date Submitted by the Author:	16-Dec-2016
Complete List of Authors:	Doyle, Gerardine; University College Dublin, College of Business<br>O'Donnell, Shane; University College Dublin<br>Quigley, Etáin; University College Dublin<br>Cullen, Kate; University College Dublin<br>Gibney, Sarah; University College Dublin<br>Levin-Zamir, D ; University of Haifa,<br>Ganahl, Kristin; Ludwig Boltzmann Institut Health Promotion Research<br>Mueller, Gabriele; Technische Universitat Dresden<br>Muller, Ingrid; University of Southampton, Department of Psychology<br>Maindal, Helle; Aarhus University<br>Chang, Wushou; School of Public Health, Taipei Medical University; Department of Occupational Medicine, Shuang Ho and Taipei Medical University Hospital<br>Van Den Broucke, Stephan; Universite catholique de Louvain, Psychological sciences Research Institute
**Primary Subject Heading**:	Health economics
Secondary Subject Heading:	Diabetes and endocrinology, Health policy, Health services research
Keywords:	Time Driven Activity Based Costing, Health literacy, Cost, Diabetes Mellitus, Type 2, Self-Management

SCHOLARONE™
Manuscripts

Patient Level Cost of Diabetes Self-Management Education Programmes: An International Evaluation

Author and Co-author's names:

Doyle, G., O'Donnell, S., Quigley, E., Cullen, K., Gibney, S., Ganahl, G., Müller, G, Muller, I., Maindal, H.T,. Chang. P,. Van den Broucke,. S.

The name, postal address, e-mail, telephone, and fax numbers of the corresponding author.

Corresponding Author:
Professor Gerardine Doyle:

University College Dublin
UCD College of Business
Q210, Lochlainn Quinn School of Business
Belfield
Dublin 4
Ireland
gerardine.doyle@ucd.ie
Tel: +353 1 716 4717
Fax: +353 1 716 4767

The full names, institutions, city, and country of all co-authors.

Names	Institutions	City	Country
Doyle, Gerardine	University College Dublin	Dublin	Ireland
O'Donnell, Shane	University College Dublin	Dublin	Ireland
Quigley, Etáin	University College Dublin	Dublin	Ireland
Cullen, Kate	University College Dublin	Dublin	Ireland
Gibney, Sarah	University College Dublin	Dublin	Ireland
Levin-Zamir, Diane	Clalit Health Services	Tel-Aviv	Israel
Ganahl, Kristin	Ludwig Boltzmann Institute	Vienna	Austria
Müller, Gabrielle	Technische Universität Dresden	Dresden	Germany
Muller, Ingrid	University of Southampton	Southampton	United Kingdom
Maindal, Helle Terkildsen	Aarhus University	Aarhus	Denmark
Chang, Peter	Taipei Medical University	Taipei	Taiwan
Van den Broucke, Stephan	Université Catholique de Louvain	Louvain	Belgium

Key words: Diabetes Mellitus Type 2, Self-management, Cost, Time Driven Activity Based Costing, Health Literacy

Word Count - excluding title page, references, figures and tables = 4,427.

For peer review only - http://bmjopen.bmj.com/site/about/guidelines.xhtml

Abstract

Objectives: The objective of this study was to examine the value of Time Driven Activity Based Costing (TDABC) in understanding the process and costs of delivering Diabetes Self-Management Education programmes (DSME) in a multi-country comparative study.

Setting: Outpatient settings in five European countries (Austria, Denmark, Germany, Ireland, United Kingdom) and two countries outside Europe, Taiwan and Israel.

Participants: Providers of DSME programmes across participating countries (N=16) including health care professionals, administrators and patients taking part in DSME programmes.

Primary and secondary measures: Primary Measure: Time spent by providers in the delivery of DSME and resources consumed in order to compute programme costs. Secondary measures: self-report measures of behavioural self-management and diabetes disease/health related outcomes.

Results: We found significant variation in costs and the processes of how DSME programmes are provided across and within countries. Variations in costs were driven by a combination of price variances, mix of personnel skill and efficiency variances. Higher cost programmes were not found to have achieved better relative outcomes. The findings highlight the value of TDABC in calculating a patient level cost and potential of the methodology to identify process improvements in guiding the optimal allocation of scarce resources in diabetes care, in particluar for DSME that is often underfunded.

Conclusions: This study is the first to measure programme costs using estimates of the actual resources used to educate patients about managing their medical condition and is the first study to map such costs to self-reported behavioural and disease outcomes. The results of this study will inform clinicians, managers and policy makers seeking to enhance the delivery of DSME programmes. The findings highlight the benefits of adopting a TDABC approach to understanding the drivers of the cost of DSME programmes in a multi-country study to reveal opportunities to bend the cost curve for DSME.

Article Summary

Strengths and limitations of this study

- Time Driven Activity Based Costing (TDABC) has rarely been applied to care pathways within non-acute settings and as such offers a novel perspective on understanding the costs of providing chronic disease self-management education.
- This is the first multi-country study to compare the costs of DSME across a number of countries within the EU and Asia.
- Outcomes of programme participation were measured through self-reported changes, making it difficult to establish if any clinical improvement occurred. Future studies should combine TDABC analysis with clinical outcomes to further assess value in DSME.

For peer review only - http://bmjopen.bmj.com/site/about/guidelines.xhtml

1.0 Introduction

Type 2 diabetes mellitus is one of the major public health threats of the 21st century, currently affecting approximately 59.8 million people within Europe and 415 million worldwide [1]. Further, it has been reported that diabetes medical care accounts for a disproportionate allocation of health service resources across the western world [1]. Developing the self-care capacity of patients is critical to determining optimal clinical, behavioural and psychosocial outcomes and therefore reducing costs [2]. Diabetes self-management education (DSME) has been shown to improve patient outcomes by reducing the onset and/or advancement of diabetes related complications; by improving quality of life; strengthening self-efficacy and personal empowerment; assisting with the development of healthy coping skills; and by reducing diabetes related distress and depression [3].

The operation and delivery of DSME varies across the international landscape. They can be either professionally led or peer led. Further, they can be group based, individually based, and increasingly IT based. In addition, DSME curricula, duration and delivery may vary substantially, both within and between countries[4]. It is well established that DSME programmes are a low cost intervention per patient and cost effective from a payer perspective. For example, a recent report published by *The Center For Health Law And Policy Innovation* (Harvard Law School) argues that cost savings can be made by public and private insurers in the United States if cost sharing were eliminated and DSME were provided free of charge to patients [5]. However, little research has explored why the costs of running such interventions vary across different health care systems and jurisdictions, or why these costs may differ. This study addresses this gap in the prior literature.

Indeed most of the economic analyses has thus far focused on establishing the cost effectiveness of DSME by comparing the cost of programmes relative to their clinical effectiveness. Such evaluations are usually based on economic modelling, carried out alongside randomised control trials and the findings typically suggest that DSME interventions are cost effective relative to usual care [6-12]. Despite this, a recent report published by the *Health Information and Quality Authority* (HIQA) [13] in Ireland highlights the large degree of heterogeneity in the methodological approaches used in such economic evaluations. This, in turn, makes results difficult to compare in any meaningful way. In addition, these approaches tend to focus solely on overall cost of running the programmes and neglect to explore potential mechanisms through which DSME programmes could be made more efficient whilst also maintaining high standards of effectiveness. Furthermore, the majority of studies are based on interventions within a US population, and as such may not be generalizable across differing health care, social and cultural contexts.

For peer review only - http://bmjopen.bmj.com/site/about/guidelines.xhtml

This study seeks to address these existing gaps in the literature through an economic evaluation of DSME delivery across a number of EU and non-EU countries, namely Austria, Denmark, Germany, Ireland, Israel, Taiwan and the UK. The selection of these countries was based on access of the Diabetes Literacy Consortium[1] to local knowledge and networks required to carry out the necessary fieldwork. These countries also represent a diversity of contrasting approaches to the delivery of DSME tailored to each country [4]. The findings are part of a wider study conducted by the Diabetes Literacy Consortium, the overall purpose of which was to examine the (cost)-effectiveness of diabetes education across Europe, Israel, Taiwan and the US[2]. The objective of this study is to examine the value of Time Driven Activity Based Costing (TDABC) in understanding the process and costs of delivering Diabetes Self-Management Education programmes (DSME) in multiple countries and sites (7 countries, 16 sites) and to identify potential process improvements in the delivery of such programmes to reveal opportunities to bend the cost curve for DSME.

2.0 Method

A Time Driven Activity Based Costing (TDABC) method was used to map the process of programme delivery and to derive patient level costs[14 15]. TDABC has been developed as a viable costing method with the health care sector by Kaplan and Porter [16 17] enabling detailed patient level costs to be computed alongside the identification of possible process improvements resulting in potential cost savings. TDABC is particularly compatible with type 2 diabetes care as the model can be applied to diverse care pathways, particularly chronic disease management. Adopting a TDABC approach in this study therefore gave increased visibility into areas of DSME delivery where process improvements and cost savings could be made, while still maintaining a high quality of patient education. Examples of the application of TDABC have been mostly confined to medical conditions and to acute clinical settings[17-19]. This study seeks to add to this body of knowledge on the costs of care within outpatient environments through identifying the patient level cost of a variety of DSME programmes both *cross*-nationally and *Intra*nationally[20]. A primary objective was to provide a robust costing framework within which future studies could include clinical and quality of life outcomes to determine the economic value added to diabetes care through the use of DSME.

The TDABC method involves seven steps [16] 1) select the medical condition and/or patient population to be examined; 2) define the care value chain; 3) develop process maps of each activity in patient

[1] The Diabetes Literacy Consortium represents a group of countries funded by the European Commission under the Seventh Framework research programme (Grant Agreement Number: 306186).
[2] http://www.diabetesliteracy.eu

For peer review only - http://bmjopen.bmj.com/site/about/guidelines.xhtml

care delivery; identify the resources involved and any supplies used for the patient at each process step; 4) obtain time estimates for each process step; 5) estimate the cost of supplying each patient care resource; 6) estimate the practical capacity of each resource provided and calculate the capacity cost rate; 7) compute the total costs over each patient's cycle of care. By constructing a sequential activity and process step map and care value chain the researcher can analyse the maps/care pathway for duplication. These areas can then be explored further to establish if changes in the pathway would add value by maintaining/increasing the level of care to the patient whilst decreasing the economic cost to the overall health care system in terms of providing DSME programmes.

Each international study team identified the care value pathway in their country and collected the activity and time data related to the care value pathway, through qualitative semi-structured interviews of health care providers from each education programme (N=16). These included physicians, nurses, educators and managers. This information was then entered into an aggregated, de-identified database for analysis. All study teams then collected resource and financial data, utilising a standardised costing worksheet related to the activities, which were then incorporated into the aggregated database for analysis. This methodology was applied to each education programme across each country included in the study. The topic guide was developed in the English language and was then subsequently translated into the local language by the local research teams in each of the participating countries.

All activities associated with the DSME pathway were entered into an aggregated Excel database. All activity and time data was collected via the survey instrument, and cost estimates were assigned to these activity variables using financial data provided by the local provider organizations.

DSME programme costs were derived specifically from the cost of performing each activity in the delivery of the programme. All cost data was entered into activity spread-sheets and therefore the data collected did not contain any information relating to identifiable individual service providers. In the resulting database, all cost information was linked to activities and not to individuals. All activity and cost information is reported per DSME programme.

To compare the outcomes of the DSME programmes, a multi-centre observational pre-post study design was used involving diabetes patients enrolled in each of the DSME programmes. Data from the participants were collected at the beginning of the programme and after three to six months.

For peer review only - http://bmjopen.bmj.com/site/about/guidelines.xhtml

The programmes included in the study were existing programmes using five different modes of delivery: individual education in one-on-one sessions, beyond routine treatment provided, group education, self-help groups, or a combination of some of the above delivery modes. The content of peer-led and structured DSME programmes was not comparable. Therefore the two peer-led programmes were excluded from our data analysis.

2.1 Study Sample

To be selected for inclusion, programmes had to: (1) target type 2 diabetes patients; (2) be conducted among the general patient population rather than tailored to the needs of a specific age cohort, needs or gender group; (3) include (but not be limited to) newly diagnosed patients; (4) be stand-alone programmes rather than an add-on to another programme or part of a wider curriculum with (multiple) parallel programs; (5) admit new patients during the time of the baseline data collection. The study sample size was driven by the number of programmes involved in the delivery of the specific DSME programmes in each country. Costs were collected for the duration of each programme, which ranged in duration from one day to those spanning a 12-month timeframe.

2.2 Analytic approach

The Time Driven Activity Based Costing (TDABC) model was utilised to calculate a cost per programme. Significant variations in programme costs prevailed despite broadly similar programme curricula across countries and programmes. Data collected revealed significant variation in number of education hours across the programmes, number and types of personnel delivering the programmes, practitioner hours and number of participating patients.

Two concepts and measures were drawn upon to develop the TDABC model[16], the unit cost of supplying capacity and the time it takes to undertake an activity. First, the model was used to calculate the cost of all the resources supplied to each programme. This included personnel, supervision and overheads including rent, equipment and software and insurance. The total cost was then divided by the actual capacity in order to calculate the cost rate. Second, the capacity cost rate was used to assign the programme costs to objects by estimating demand on the resource. Two variables were estimated: the capacity cost rate for the programme and the capacity use by each patient. The capacity cost rate was calculated by:

$$\text{Capacity Cost Rate} = \frac{\text{Cost of Capacity Supplied}}{\text{Practical Capacity of Resource Supplied}}$$

For peer review only - http://bmjopen.bmj.com/site/about/guidelines.xhtml

Practical capacity was used as the denominator in the capacity cost rate equation. Estimating the practical capacity required two time estimates which were gathered from Human Resources and other administrative records: the total number of days that each employee actually worked each year; the total number of hours per day that the employee was available for work. Practical capacity was calculated as 80% of this working time [16]. Therefore 20% was attributed to breaks, training and annual leave. This was applied to all countries to ensure consistency and comparability of the computed programme costs.

In order to calculate the total cost of each DSME programme per patient, the capacity cost rates (including associated support costs) for each resource used was multiplied by the amount of time attributed to each patient. This calculation was based on the number of patients enrolled at the outset of the programme. The total cost of each programme per patient was the sum of all the costs across all the processes within the DSME programme. The costs were collected in the local currency and then expressed in international dollar to ensure comparability of the costs by using the Purchasing Power Parity conversion factors, to control for different standards of living, different wage levels across countries and for the particular exchange rate.

As suggested by Erhun et al. we performed a quantitative investigation of the differences in consumption and pricing of labour resoucres using cost variance analysis on labour costs. This analysis enabled us to quantitatively discern differences between processes at selected sites. The cost difference can be divided into two effects, a price (due to different capacity cost rates of labour resource (CCR)) and a quantity variance (due to different use of the labour resource across the sites). We performed this variance analysis to understand the differences in consumption and pricing of labour resources and to understand the drivers of cost variation across capacity cost rate variances, mix of personnel and efficiency variances[17]

To understand the association between programme cost and health outcomes achieved, we mapped the cost per programme to self-reported patient outcomes. Due to the significant difference in access to clinical data across the participating countries in this study, it was not possible to collect comparable clinical data for each country. Therefore comparable data was collected to measure outcomes at behavioral and disease/health outcome levels for existing diabetes self-management programs. Health outcome data was collected at three levels; individual diabetes self-management disposition, behaviour and disease/health related outcomes. (The outcome framework employed in this study is summarised in the supplemental file attached.)

For peer review only - http://bmjopen.bmj.com/site/about/guidelines.xhtml

For peer review only

A statistically significant improvement was found for six behavioral outcomes (general diet, exercise, medication use, problem areas in diabetes, foot care and appraisal of diabetes) and three disease/health outcomes (BMI, health related quality of life and affective well-being) in the total sample. We analyse these self-reported patient outcomes to the cost per programme.

3.0 Results

Findings highlight that the programmes included in this study provide similar educational content when delivering diabetes education. Further, we found similar changes in self-reported behavioural and disease outcomes across programmes. This suggests that factors other than educational content drives cost variation across programmes and despite reported cost variation, outcomes appear broadly similar. The cost difference between two sites can be analysed into two effects: a price variance due to different capacity cost rates of resource and a quantity variance due to different use of resource:

$$\Delta_{1,2} = \sum_{i=1}^{N_L} q_1^i \times r_1^i - \sum_{i=1}^{N_L} q_2^i \times r_2^i$$

Figure 1 presents the price variance across the sites[3].

Insert Figure 1 here.

There are a number of factors which were found to influence cost variation. Firstly, programmes differed in duration and hours of practitioner time spent on each programme delivery. This reflects the efficiency variance due to different quantities of total personnel used. For example, Figure 2 highlights that the 'Ireland 2' programme utilises 78 hours of personnel, whereas 'Austria 3' only uses 5.25 hours of personnel time, yet patient self-reported outcomes are broadly similar. This suggests that total personnel time is a strong cost driver but not an outcome driver. This efficiceny variance across two sites is expressed as:

$$= \left(\sum_{i=1}^{N_L} r_2^i \times \frac{q_2^1}{Q_2} \right) \times (Q_1 - Q_2)$$

[3] For each Figure 1-6 the associated data is included in Supplemental Files attached.

For peer review only - http://bmjopen.bmj.com/site/about/guidelines.xhtml

Insert Figure 2 here.

Secondly, mix of personnel skill used in providing the education is a cost driver. For example, the high salary cost for a consultant physician in Germany and social worker cost in Israel (Figure 3) did not produce any significant improvement in patient self-reported outcomes. These findings suggest that personnel skill used is a strong cost driver but does not significantly alter patient self-reported outcomes. When comparing two sites this mix variance is measured as follows:

$$= \left(\sum_{i=1}^{N_L} \left(\frac{q_1^i}{Q_1} - \frac{q_2^i}{Q_2} \right) \times r_2^i \right) \times Q_1$$

Insert Figure 3 here.

Figure 3 highlights that for most countries the salaries and practical capacity cost rates for those who provide DSME are broadly similar. The exceptions to this include Germany who use a consultant physician for part of the education programme, Israel who have a high salary scale for social workers and Taiwan who have a low salary rate for the hospital nurses providing patient education.

Thirdly, the number of patients who attended each programme was a strong per-patient cost driver (Figure 4) the more patients who attended the programme the lower the per-patient cost.

Insert Figure 4 here.

Taking total cost per programme, the median programme was identified as Israel Programme 1. The key cost drivers identified were then compared to this base programme to explore the behaviour of these variances. Figure 5 summarises this comparison with the base country and reveals that there is a non-linear relationship between the cost of a programme and each of the key cost drivers, practitioner hours used, the practical capacity rate of the skill mix used and the number of patients participating on the programmes. This reveals the complexity of the cost behaviours and of the cost

For peer review only - http://bmjopen.bmj.com/site/about/guidelines.xhtml

variations between the programmes despite offering similar curricula and resulting in similar health outcomes.

Insert Figure 5 here.

Figure 6 maps the health outcomes observed with the cost per programme. Very modest improvements in each of the self-reported variables were found. More significantly, there was very little variation in outcomes across each of the programmes, both within and between countries, whatever the mode of delivery, mix of personnel skill used, quantity of total personnel hours, quantity of education hours or quantity of participating patients.

Insert Figure 6 here.

4.0 Discussion

The data illustrates that Diabetes Self Management Education programmes are provided at a low cost in every country studied. The data provides evidence that while these costs are low, significant cost variations exist both within and between countries. This is due to a combination of cost variations between the programmes; the capacity cost rate, the mix of personnel delivering the education, the different quantities of total personnel used and the number of patients participating in these programmes. This is the first time that such multi-country comparative data has been collected.

The variance analysis performed surrounding costs and outcomes illustrates total personnel hours as a strong cost driver (Figure 2). Practitioners such as nurses and diabetes nurse specialists can produce similar outcomes to physicians but at a lower salary and practical capacity cost. This is likely to be a more effective use of resources, particularly in relation to optimizing use of personnel at their level of expertise. Further research is needed to explore the most appropriate level of expertise required to deliver the programme for optimal patient health outcomes. For example, instead of having a consultant physician or a Clinical Nurse Specialist delivering the education programmes it may be more appropriate to have well trained experienced nurses or the equivalent performing this

For peer review only - http://bmjopen.bmj.com/site/about/guidelines.xhtml

role. A pilot study conducted by Kaplan et al. at The University of Texas Cancer Centre revealed that matching clinical skills to the processes led in a 16% reduction in process time, a 12% decrease in costs for technical staff, and a 67% reduction in costs for professional staff[16]. However clinical outcomes, in addition to behavioural and psychosocial outcomes, are necessary to determine fully whether the educators' level of expertise really influences all DSME health outcomes[16].

In some countries the cost of the same programme varied by site. For these programmes, we observed significant variation in administrative hours and this was not associated with the number of participating patients. This finding is similar to that of Munoz et al. who used TDABC in a cost-effectiveness analysis of a red blood cell salvage post total-knee arthroscopy in the US, Switzerland and Austria and suggest that tighter control of administrative costs may reduce what appear to be non-value added hours for the patients[21].

Integrating data on the number of patients participating on each programme (Figure 4) with the outcome data suggests that the number of patients in attendance did not impact on patient self-reported outcomes. These findings suggest that there is room for cost savings in DSME regarding the amount of hours of education provided, who provides the education and the number of patients in attendance at each programme, without negatively impacting patients self-reported outcomes.

A number of learnings emerged from this study: firstly, all programme curricula covered similar topics, this suggests that there is a shared consensus on what information requires dissemination and highlights that variation relates to process delivery rather than curricula; secondly, the administrative burden on programmes varies greatly and as such is an area of programme development which requires planning and streamlining; thirdly, the skill mix of professionals delivering the programme varies greatly suggesting a lack of empirical knowledge surrounding the most effective educator; fourthly, the duration and hours of education varies significantly across sites, again highlighting a lack of consensus in terms of the most efficacious course construct; and finally, such cost variation exists across sites despite the programme content being broadly similar. The granular mapping of the DSME programmes and the derivation of a cost per programme is the first step in generating a better understanding of the DSME arena internationally.

Analysis of the self-reported outcome data found that these outcomes were similar irrespective of the education programme or the country (albeit that the sample size was small and the standard deviation high). Across each programme, a statistically significant improvement was found for six

For peer review only - http://bmjopen.bmj.com/site/about/guidelines.xhtml

behavioral outcomes (general diet, exercise, medication use, problem areas in diabetes, foot care and appraisal of diabetes) and three disease/health related outcomes (BMI, health related quality of life and affective well-being).

The costing and provision of DSME is at an early stage of development globally with limited empirical knowledge of the most efficient and effective mode of delivering DSME. Thus this study has gone some way to remedying this problem whereby it has outlined a bottom up/patient level cost using estimates of the actual resource costs used to educate patients through self-management programs and therefore a more accurate cost than heretofore of providing various education programmes. Thus, it has provided a first layer of information, which in the future will be required to establish whether this model of care/intervention can add value to the health care system once clinical effectiveness outcomes have been determined for each programme. Storfjell et al., show that the application of TDABC in the context of nursing care can facilitate the identification and elimination of non-value added time (NVA) and related the increase in time spent on psychosocial intervention, support and patient education[22]. However, there is a long way to go, whereby clinical and Quality of Life outcomes are required to measure the effectiveness of DSME programmes before a thorough understanding of their added value to patients can be estimated. The methods and results of the current study will inform future research to achieve a better understanding of the added value derived from providing DSME interventions. We suggest that future studies include a rigorous collection of clinical outcomes pre and post DSME.

5.0 Limitations

The TDABC method is a relatively new method in terms of healthcare costing and to the best of the authors' knowledge has yet to been applied to investigate the costs of a health education intervention. As a result there were limited guidelines surrounding the collection of activity and process step data in non-acute settings and thus it was necessary for the research team to develop such a protocol that was fit-for-purpose across different international study sites. In practice, many participants were unfamiliar with the costing and activity terminology and the level of detail required on all forms of activity, for TDABC. We observed that participants appeared to provide less detail on administrative and programme preparation activity compared with education activity. This detailed information would have provided greater insight into the reasons why administrative costs were found to be so high in some countries while not in others.

For peer review only - http://bmjopen.bmj.com/site/about/guidelines.xhtml

In addition, some of the local research teams also experienced difficulties in collecting the required financial data. For example, in Belgium, the staff involved in the delivery of DSME programmes taking part in this study were unwilling to share salary information at the level of granularity that was required to compute a programme cost. For this reason, the Belgian data had to be excluded from this particular study.

The study is also limited by a lack of available clinical outcome data from each of the education programmes. While important self-reported health and psycho-social outcome data was collected in each country it was not possible to determine the clinical-effectiveness of these DSME programmes in terms of glycemic control due to the absence of any clinical measures. As Kaplan and Porter point out[16], value in health care can only be determined when there is visibility into both costs and *clinical* outcomes. Furthermore, the reliability of self-reported outcomes data was undermined by small sample sizes in each country. Secondly, self-reported measures of health behavior are susceptible to social desirability bias, and response styles can vary by culture and setting [(23, 24)]. Nonetheless, the similarity in outcomes across each of the sites regardless of the amount of money invested in each programme raises questions surrounding the value being achieved per Euro/ dollar spent.

The peer-led programmes found in Denmark and Germany were excluded from the analysis. However, they were provided at the lowest cost of Int $0.15 and Int $0.74 per patient per hour of education respectively. When self-assessed outcome data was measured for each programme, the outcomes were similar for peer-led and specialist-led programmes. We suggest that further research is needed surrounding peer-led education and measurement of associated clinical health outcomes.

6.0 Conclusion

This paper has demonstrated the variances in the cost of delivering different types of diabetes education programmes, both within and across countries in the EU and Asia. Developing cost effective lifestyle interventions to improve the diabetes knowledge and self-management skills and quality of life for patients may be an important step in preventing the onset of complications associated with type 2 diabetes. The imperative to do so from an economic perspective cannot be underestimated when consideration is given to the implications for health care systems associated with the treatment of diabetes related morbidities such as active foot disease, chronic kidney disease, retinopathy and myocardial infarction[25].

For peer review only - http://bmjopen.bmj.com/site/about/guidelines.xhtml

This study offers the first application of a TDABC approach to evaluate the cost of delivering DSME programmes and as a means of comparing the costs of running a healthcare intervention cross-nationally. It contributes to the extant literature by highlighting and describing the vast combinations and permutations of delivery of DSME curricula, practitioner hours, hours of education, mix of educators, numbers of attendees and how these variations lead to substantial cost differences. Our variance analysis revealed that the key drivers of cost variation arose from differing capacity cost rates, the mix of personnel delivering the education, the different quantities of total personnel used and the number of patients participating in these programmes. In the process, we identified how there could be potentially unnecessary process steps that, if eliminated, could lead to cost savings in the delivery of DSME programmes, including vast differences in administration time, and exploring the mix of personnel skill alongside the total personnel time used.

While it is already established that diabetes education is a low cost intervention and is cost-effective, given the sheer numbers of education programmes that need to be made available to meet the demands resulting from increasing levels of diabetes worldwide, even small process improvements could lead to overall cost savings for healthcare providers. Future studies focusing on the cost-effectiveness of healthcare interventions may consider adopting TDABC principles and variance analysis as a means of identifying efficiencies in other chronic disease education programmes.

The study has highlighted the strengths of TDABC as a method of bottom up costing in outpatient care and recommends utilising this method in future studies so as to allow for a comprehensive literature to develop in the area, enabling comparative studies to be performed. By developing such literature a comprehensive understanding of the cost of patient education programmes can be developed and compared cross nationally and across time. Health care practitioners and educators who wish to convince policy makers and health insurers to reimburse the cost of DSME delivery can adopt a TDABC approach in order to demonstrate that such programmes are run efficiently and effectively especially when combined with measures of consequent clinical health outcomes to represent value for money.

Acknowledgements

This article is part of the Diabetes Literacy project supported by grant FP7-Health-2012-Innovation-1/306186 of the European Commission. The authors would like to thank the partners of the Diabetes

For peer review only

Literacy Consortium for contributing to data collection with participants and providers of DSME programs in their respective countries.

The Diabetes Literacy Consortium consists of the following members:

Université Catholique de Louvain, Belgium: Stephan Van den Broucke, Gerard Van der Zanden, Marie Housiaux, Louise Schinckus.

Technische Universität Dresden, Medical Faculty, Germany: Peter Schwarz, Gabriele Mueller, Henna Riemenschneider, Sarama Saha.

University College Dublin, Ireland: Gerardine Doyle, Shane O'Donnell, Etain Quigley, Kate Cullen, Sarah Gibney

Ludwig Boltzmann Institute for Health Promotion Research, Austria: Jürgen Pelikan, Florian Röthling, Kristin Ganahl, Sandra Peer.

Maastricht University, Department of International Health, The Netherlands: Helmut Brand, Kristine Sörensen, Timo Clemens, Marjo Campmans

University of Southampton, UK: Lucy Yardley, Ali Rowsell, Ingrid Muller, Victoria Hayter

Clalit Health Services, Israel: Diane Levin-Zamir, Ziv Har-Gil.

University of California at San Francisco, USA: Dean Schillinger, Courtney Lyles, Lina Tieu.

Taipei Medical University, Taiwan: Peter Chang, Candy Kuo, Alice Lin, Duong Van Tuyen, Becky Sun.

Aarhus University, Denmark*:* Helle Terkildsen Maindal, Jill Rowlands, Ulrik Martensen.

We also thank all the medical and educational services from the eight participating countries who contributed to the data collection.

Contributorship statement GD initially proposed the study. GD and SG specified the methodology. KC carried out the cost analysis. All authors contributed to the protocol design, data collection and analysis plan. EQ and SO'D wrote the initial manuscript, and all authors contributed to improving the manuscript. All authors approved the final manuscript.

Competing interests None declared.

Ethics Approval All methods were approved by the SVUH group Research and Ethics Committee, by the Research Ethics Committee of the Office of Research Ethics, University College Dublin and by the relevant local ethics committees in each jurisdiction and each study site where the study was carried out.

Funding This study is part of the Diabetes Literacy project supported by grant FP7-Health-2012-Innovation 1/306186 of the European Commission.

For peer review only

Data sharing statement The Excel spreadsheets showing how the individual activity costs were aggregated are available should they be requested.

References

1. International Diabetes Federation. IDF Diabetes Atlas. 7 ed ed. Brussels: International Diabetes Federation, 2015.
2. Peyrot M, Burns KK, Davies M, et al. Diabetes Attitudes Wishes and Needs 2 (DAWN2): A multinational, multi-stakeholder study of psychosocial issues in diabetes and person-centred diabetes care. Diabetes Research and Clinical Practice 2013;**99**(2):174-84.
3. Powers MA, Bardsley J, Cypress M, et al. Diabetes Self-management Education and Support in Type 2 Diabetes A Joint Position Statement of the American Diabetes Association, the American Association of Diabetes Educators, and the Academy of Nutrition and Dietetics. The Diabetes Educator 2015;**41**(4):417-30.
4. Saha S, Müller G., Riemenschneider H, et al. Compendium of Diabetes Self-Management Education Programs in the European Union Member States, Israel, Taiwan and the USA. Version 1.1, Dresden, 2015.
5. Garfield K, Downer S, Rosenberg A, et al. Reconsidering Cost Sharing for DSME: Recommendation for Policy Reform. Massachusetts: Harvard, 2015.
6. Gillespie P, O'Shea E, Paul G, et al. Cost effectiveness of peer support for type 2 diabetes. International journal of technology assessment in health care 2012;**28**(01):3-11.
7. Brown 3rd H, Wilson KJ, Pagán JA, et al. Cost-effectiveness analysis of a community health worker intervention for low-income Hispanic adults with diabetes. Prev Chronic Dis 2012;**9**:E140.
8. Brownson CA, Hoerger TJ, Fisher EB, et al. Cost-effectiveness of Diabetes Self-management Programs in Community Primary Care Settings. The Diabetes Educator 2009;**35**(5):761-69.
9. Kuo S, Bryce CL, Zgibor JC, et al. Cost-Effectiveness of Implementing the Chronic Care Model for Diabetes Care in a Military Population. Journal of Diabetes Science and Technology 2011;**5**(3):501-13.
10. Jacobs-van der Bruggen MA, van Baal PH, Hoogenveen RT, et al. Cost-effectiveness of lifestyle modification in diabetic patients. Diabetes Care 2009;**32**(8):1453-58.
11. Dall TM, Roary M, Yang W, et al. Health care use and costs for participants in a diabetes disease management program, United States, 2007–2008. Prev Chronic Dis 2011;**8**(3):A53.
12. Molsted S, Tribler J, Poulsen PB, et al. The effects and costs of a group-based education programme for self-management of patients with Type 2 diabetes. A community-based study. Health Education Research 2012;**27**(5):804-13.
13. Health Information and Quality Authority. Health technology assessment of chronic disease self-management support interventions. Dublin: HIQA, 2015.
14. Kaplan R, Anderson SR. Time-driven activity-based costing: a simpler and more powerful path to higher profits. Harvard business press 2013
15. Kaplan RS, Anderson SR. The innovation of time-driven activity-based costing. Journal of cost management 2007;**21**(2):5-15.
16. Kaplan RS, Porter ME. How to solve the cost crisis in health care. Harvard Business Review 2011;**89**(9):46-52.
17. Erhun F, Mistry B, Platchek T, et al. Time-driven activity-based costing of multivessel coronary artery bypass grafting across national boundaries to identify improvement opportunities: study protocol. BMJ open 2015;**5**(8):e008765.
18. Akhavan S, Ward L, Bozic KJ. Time-driven Activity-based Costing More Accurately Reflects Costs in Arthroplasty Surgery. Clinical Orthopaedics and Related Research® 2016;**474**(1):8-15.

For peer review only - http://bmjopen.bmj.com/site/about/guidelines.xhtml

19. Kaplan AL, Agarwal N, Setlur NP, et al. Measuring the cost of care in benign prostatic hyperplasia using time-driven activity-based costing (TDABC). Healthcare 2015;**3**(1):43-48.
20. Demeere N, Stouthuysen K, Roodhooft F. Time-driven activity-based costing in an outpatient clinic environment: Development, relevance and managerial impact. Health Policy 2009;**92**(2–3):296-304.
21. Muñoz M, Campos A, Martín-Montañez E, et al. Could cost-effectiveness of postoperative shed blood salvage after elective knee arthroplasty be improved? Transfusion 2013;**53**(6):1372-73.
22. Storfjell JL, Ohlson S, Omoike O, et al. Non-Value-Added Time: The Million Dollar Nursing Opportunity. Journal of Nursing Administration 2009;**39**(1):38-45.
23. Herbert JR, Yunsheng M. Clemow L., Ockene IS. Saperia G. Stanek III EJ, Merriam PA, Ockene JS: Gender differences in social desirability and social approval bias in dietary self-report, *American Journal of Epidemiology*, 146:1046-1055, 1997.
24. Kristal A. Andrilla CH. Koepsell TD. Diehr PH.Cheadle A: Dietary assessment instruments are suspectible to intervention-associated resposne set bias, *Journal of the American Dietetic Association,* 98: 40-42, 1998.
25. Seuring T, Archangelidi O, Suhrcke M. The Economic Costs of Type 2 Diabetes: A Global Systematic Review. PharmacoEconomics 2015;**33**(8):811-31.

For peer review only

For peer review only - http://bmjopen.bmj.com/site/about/guidelines.xhtml

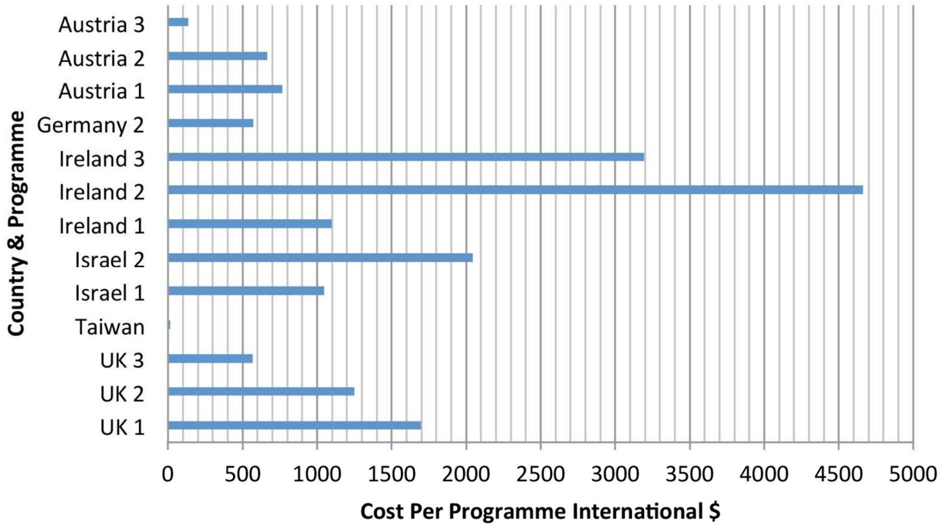

Figure 1: Cost Per Programme in International Dollars
Figure 1
145x84mm (300 x 300 DPI)

For peer review only

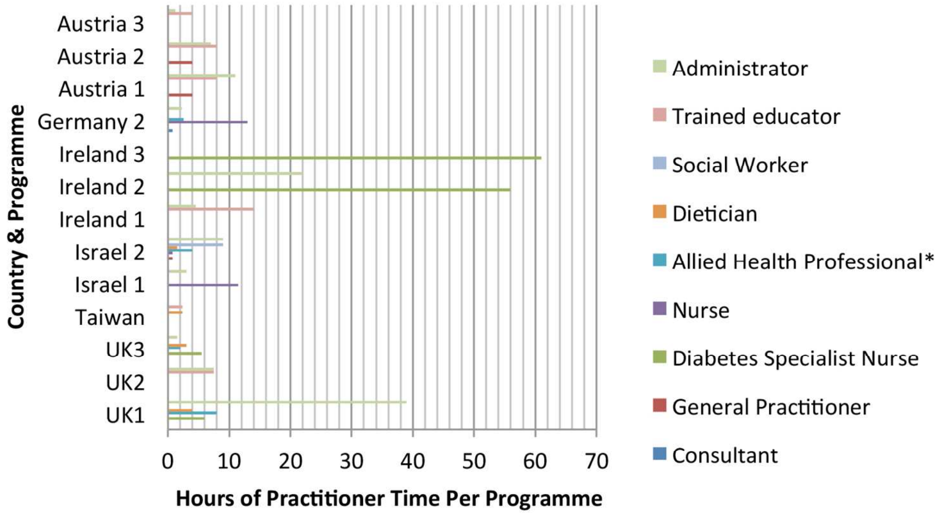

Figure 2: Hours of Practitioner Time Per Programme
*Allied Health Professional = podiatrist, physiotherapist, health promoter, sore assistant, laboratory scientist, social worker

Figure 2
145x83mm (300 x 300 DPI)

For peer review only

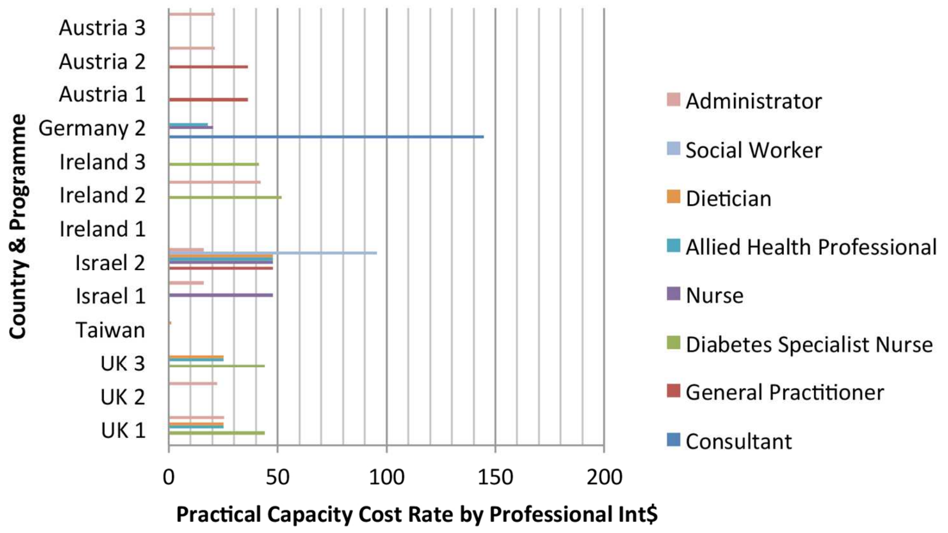

Figure 3: Practical Capacity Cost Rate by Professional Int $
Figure 3
145x84mm (300 x 300 DPI)

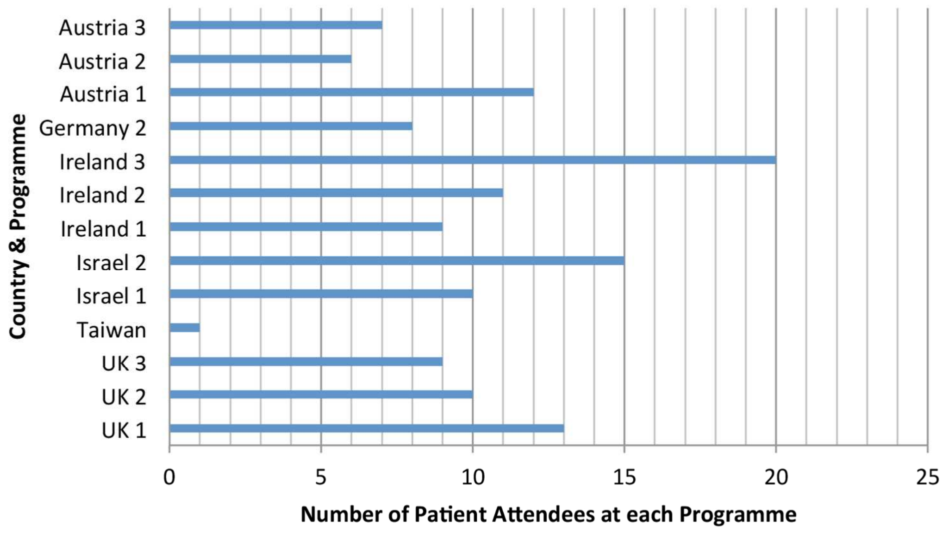

Figure 4: Number of Patient Attendees at each Programme

Figure 4
145x84mm (300 x 300 DPI)

For peer review only - http://bmjopen.bmj.com/site/about/guidelines.xhtml

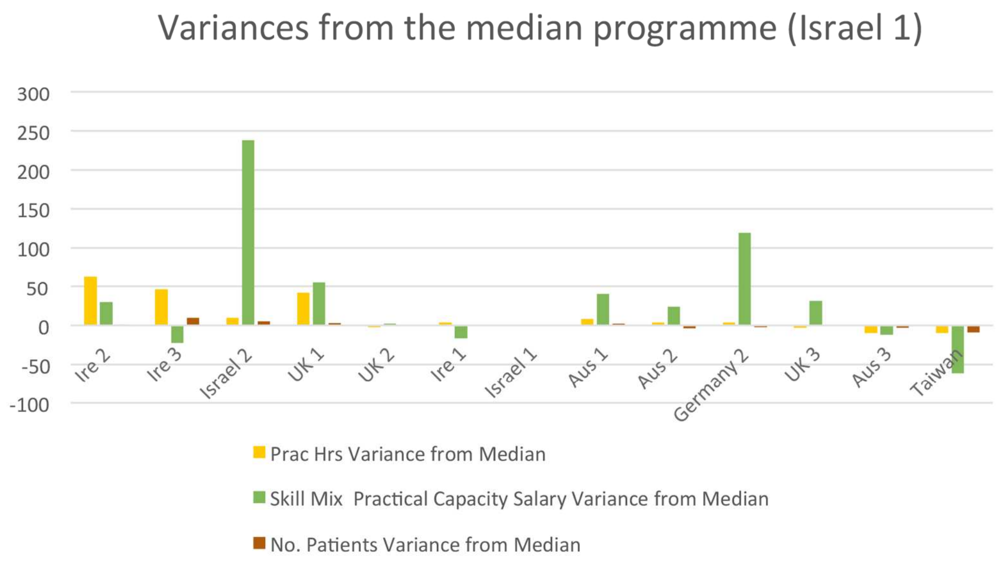

Figure 5: Variances from the median programme (Israel 1) – variances in practitioner hours, practical capacity rate and number of patients
Figure 5
145x84mm (300 x 300 DPI)

For peer review only - http://bmjopen.bmj.com/site/about/guidelines.xhtml

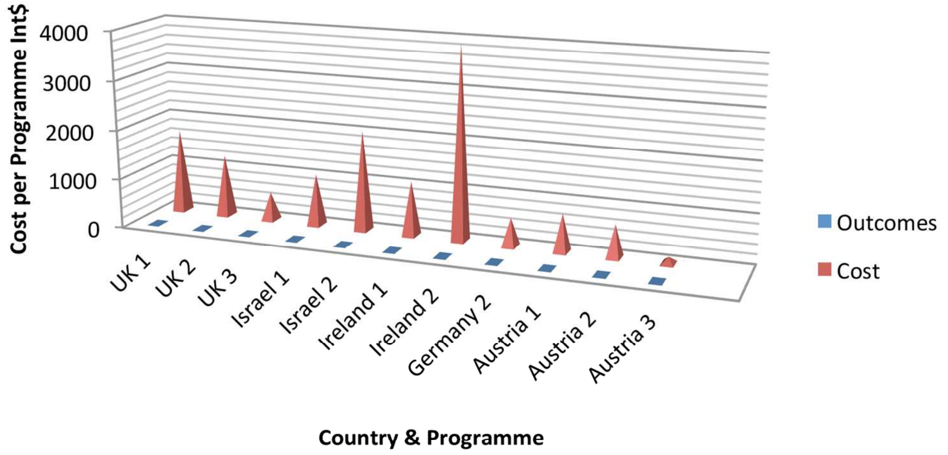

Figure 6: Cost Per Programme (Int$) and Self-Reported Patient Outcomes

Figure 6
145x84mm (300 x 300 DPI)

For peer review only - http://bmjopen.bmj.com/site/about/guidelines.xhtml

Data to Support Figures 1-5

**Programme**	Total Cost per-programme (home currency)	Total Cost per-programme (Int$)
UK 1	£1201.83	1697.50
UK 2	£883.60	1248.02
UK 3	£402.03	567.83
Taiwan	227.71NTD	14.01
Israel 1	4196.90 INS	1046.61
Israel 2	8196.08 INS	2043.91
Ireland 1	€923.38	1099.26
Ireland 2	€3918.31	4664,65
Ireland 3	€2682.40	3193.33
Germany 2	€451.52	573.72
Austria 1	€619.68	766.93
Austria 2	€536.28	663.71
Austria 3	€109.97	136.10

Figure 1 Data: Cost Per Programme (Salary and Overheads)

For peer review only

Cost Driver 1: Number of Practitioner Hours Spent on each programme

**Staff (hrs)**<br>**Programme**	Consultant	GP	DSN	Nurse	AHP	Dietician	Social Worker	Trained Educator	Administrator	Total time
UK 1	-	-	6	-	8	4	-	-	39	57
UK 2	-	-	-	-	-	-	-	7.5	5.5	13
UK 3	-	-	5.5	-	2	3	-	-	1.5	12
Taiwan	-	-	-	-	-	2.4	-	2.4	-	4.8
Israel 1	-	-	-	11.5	-	-	-	-	3	14.5
Israel 2	-	.75	-	.75	4	1.5	9	-	9	25
Ireland 1	-	-	-	-	-	-	-	14	4.5	18.5
Ireland 2	-	-	56	-	-	-	-	-	22	78
Ireland 3	-	-	60.6	-	-	-	-	-	-	60.6
Germany 2	.75	-	-	13	2.5	-	-	-	2.25	18.5
Austria 1	-	4	-	-	-	-	-	8	11	23
Austria 2	-	4	-	-	-	-	-	8	7	19
Austria 3	-	-	-	-	-	-	-	4	1.25	5.25

Figure 2 Data: Number of Practitioner Hours Spent on each Programme Course

For peer review only

Cost Driver 2: Practitioner Type and Hourly Practical Capacity Salary (in Int $)

**Staff (PC Salary)** / **Programme**	Consultant	GP	DSN	Nurse	AHP	Dietician	Social Worker	Trained Educator	Administrator	Total
UK 1	-	-	44.10	-	25.24	25.24	-	-	25.44	120.02
UK 2	-	-	-	-	-	-	-	44.10	22.10	66.20
UK 3	-	-	44.10	-	25.24	25.24	-	-	-	94.58
Taiwan	-	-	-	-	-	1.26	-	1.26	-	2.52
Israel 1	-	-	-	47.73	-	-	-	-	16.03	63.76
Israel 2	-	47.73	-	47.73	47.73	47.73	95.45	-	16.03	302.40
Ireland 1	-	-	-	-	-	-	-	47.51	-	47.51
Ireland 2	-	-	51.74	-	-	-	-	-	42.15	93.89
Ireland 3	-	-	41.50	-	-	-	-	-	-	41.50
Germany 2	144.65	-	-	20.32	17.84	-	-	-	-	182.81
Austria 1	-	36.26	-	-	-	-	-	30.45	21.09/37.66	104.37
Austria 2	-	36.26	-	-	-	-	-	30.45	21.09	87.80
Austria 3	-	-	-	-	-	-	-	30.45	21.09	51.54

Figure 3 Data: Practitioner Type and Hourly Practical Capacity Salary in Int$ for comparison

For peer review only

Cost Driver 3: Number of Attendees at Each Programme

Programme	Number of participants
UK 1	13
UK 2	10
UK 3	9
Taiwan	1
Israel 1	10
Israel 2	15
Ireland 1	9
Ireland 2	11
Ireland 3	20
Germany 2	8
Austria 1	12
Austria 2	6
Austria 3	7*

Figure 4 Data: Number of Patients who Attend each Programme Course

*This programme runs for insulin and non-insulin users – insulin typically have 4 patient attendees and non-insulin typically have 9 patient attendees. We took the median number = 6.5 rounded to 7.

For peer review only

Programme	Total Cost	Price Variance from Median	Practitioner Hours	Prac Hrs Variance from Median	Skill Mix *Hourly Practical Capacity Salary*	Skill Mix Practical Capacity Salary Variance from Median	No. of Patients	No. Patients Variance from Median
Ire 2	4664	3,618	78	63	94	30	11	1
Ire 3	3193	2,147	61	46	42	-22	20	10
Israel 2	2043	997	25	10	302	238	15	5
UK 1	1697	651	57	42	120	56	13	3
UK 2	1248	202	13	-2	66	2	10	0
Ire 1	1099	53	19	4	48	-16	9	-1
Israel 1	1046	0	15	0	64	0	10	0
Aus 1	766	-280	23	8	104	40	12	2
Aus 2	633	-413	19	4	88	24	6	-4
Germany 2	573	-473	19	4	183	119	8	-2
UK 3	567	-479	12	-3	95	31	9	-1
Aus 3	136	-910	5	-10	52	-12	7	-3
Taiwan	14	-1032	5	-10	3	-61	1	-9

Median Programme = Israel 1

Figure 5 Data: Variances from the median programme (Israel 1) – variances in practitioner hours, practical capacity rate and number of patients

For peer review only

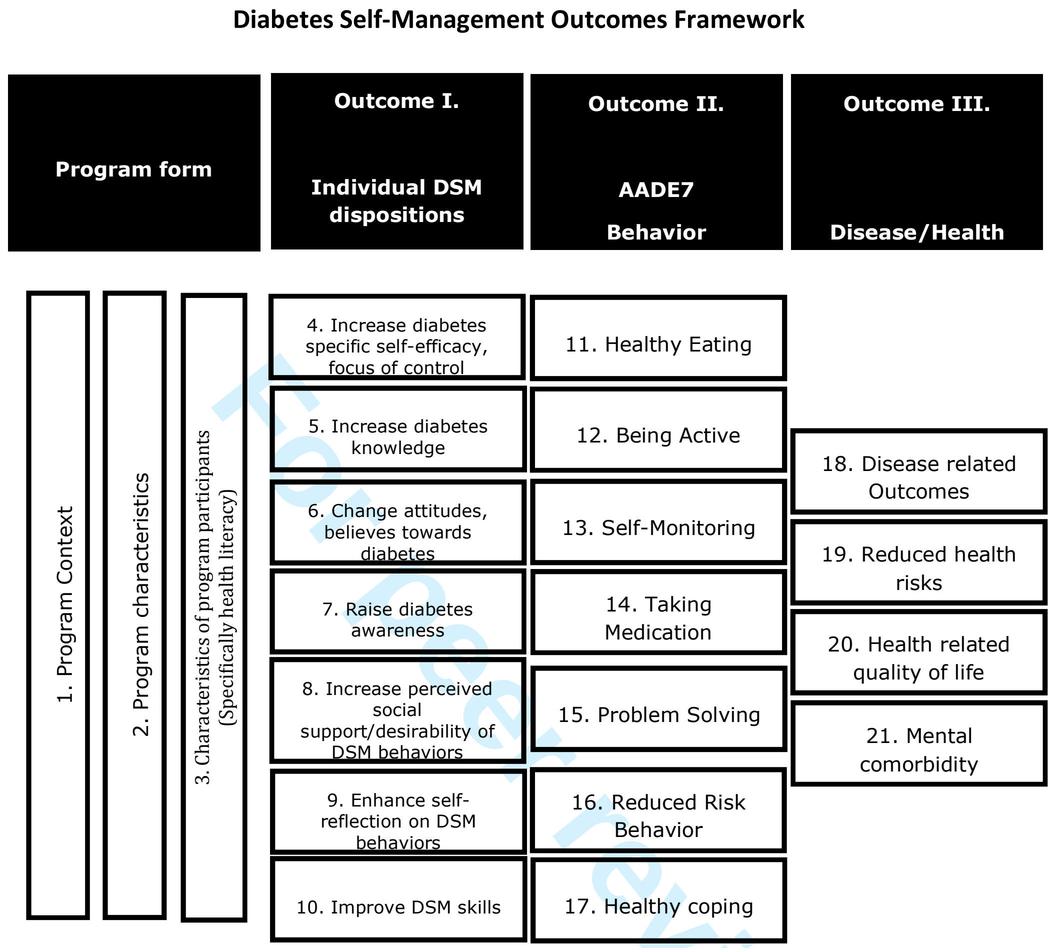

For peer review only

	N	Gen Diet	**SD**	Special Diet	**SD**	Problem Solving	**SD**	Reduced Risk Behaviour	**SD**	Health Coping	**SD**	Diabetes Health Literacy	**SD**	Fdhl	**SD**	CrDhl	**SD**
UK 1	*2*	0	**0**	1.33	**.94**	0.5	**3.54**	1.25	**0.17**	-1	**2.83**	0.07	**0.2**	0	**0.28**	0.25	**0**
UK2	*21*	0.02	**.73**	-0.21	**1.75**	0.81	**2.89**	0.11	**0.94**	1.33	**3.23**	-0.19	**0.37**	-0.3	**0.48**	-0.17	**0.59**
UK3	*27*	0.09	**1.26**	0.32	**1.01**	1.81	**4**	0.49	**0.84**	0.85	**3.45**	-0.04	**0.35**	-0.09	**0.46**	-0.01	**0.69**
GER2	*5*	0	**1.87**	0.07	**1.44**	2.6	**1.5**	0.89	**1.11**	3.14	**3.58**	0.13	**0.11**	0.11	**0.3**	0.26	**0.5**
IS1	*11*	0.59	**1.11**	-0.39	**2.33**	4.18	**5.96**	0.41	**1.4**	3.73	**3.93**	-0.03	**0.27**	0.07	**0.59**	-0.25	**0.8**
IS2	*64*	0.38	**3**	-0.22	**2.33**	0.58	**5.34**	0.23	**1.75**	1.17	**4.53**	0.07	**0.52**	0.05	**0.91**	0.04	**0.8**
IR1	*13*	0.73	**1.59**	-0.74	**1.42**	5	**4.28**	0.17	**1.71**	3	**4.08**	0.21	**0.59**	-0.12	**0.74**	0.35	**0.85**
IR2	*5*	-0.2	**0.45**	-0.07	**1.83**	4.6	**5.98**	0.55	**1.05**	3	**2.74**	0.2	**0.45**	0.36	**0.26**	-0.05	**0.59**
AUS1	*6*	0.5	**1.1**		**1.48**	1.5	**3.62**	1.38	**0.92**	3.5	**3.08**	0.07	**0.25**	-0.3	**0.37**	0.42	**0.47**
AUS2	*2*	1.5	**1.12**	1.83	**4.95**	-1.5	**4.95**	3	**1.77**	0.5	**4.95**	0.25	**0.25**	-0.2	**0.42**	0.5	**0.28**
AUS3	*5*	0.3	**.72**	0.93	**3.49**	1.2	**3.49**	0.3	**1.2**	4.4	**3.44**	-0.43	**0.42**	-0.28	**0.46**	-0.75	**0.41**

Figure 6 Data: Self-Reported Patient Outcomes (SD=Standard Deviation)

For Figure 6, General Diet was taken as one example of the health outcome data achieved when mapped with cost per programme. To include each health outcome would make Figure 6 too complex and the main finding that health outcomes were similar across all programmes would not be clear to the reader.

For peer review only

CHEERS Checklist
Items to include when reporting economic evaluations of health interventions

The **ISPOR CHEERS Task Force Report**, *Consolidated Health Economic Evaluation Reporting Standards (CHEERS)—Explanation and Elaboration: A Report of the ISPOR Health Economic Evaluations Publication Guidelines Good Reporting Practices Task Force,* provides examples and further discussion of the 24-item CHEERS Checklist and the CHEERS Statement. It may be accessed via the *Value in Health* or via the ISPOR Health Economic Evaluation Publication Guidelines – CHEERS: Good Reporting Practices webpage: http://www.ispor.org/TaskForces/EconomicPubGuidelines.asp

Section/item	Item No	Recommendation	Reported on page No/ line No
**Title and abstract**			
Title	1	Identify the study as an economic evaluation or use more specific terms such as "cost-effectiveness analysis", and describe the interventions compared.	p.4/ 131
Abstract	2	Provide a structured summary of objectives, perspective, setting, methods (including study design and inputs), results (including base case and uncertainty analyses), and conclusions.	p.2/ 50-78
**Introduction**			
Background and objectives	3	Provide an explicit statement of the broader context for the study.<br>Present the study question and its relevance for health policy or practice decisions.	p.4/ 138-142
**Methods**			
Target population and subgroups	4	Describe characteristics of the base case population and subgroups analysed, including why they were chosen.	p.6/ 204-211
Setting and location	5	State relevant aspects of the system(s) in which the decision(s) need(s) to be made.	p.2/ 54-55
Study perspective	6	Describe the perspective of the study and relate this to the costs being evaluated.	p.5-6/ 188-201
Comparators	7	Describe the interventions or strategies being compared and state why they were chosen.	p.6/ 204-211
Time horizon	8	State the time horizon(s) over which costs and consequences are being evaluated and say why appropriate.	p.6/ 211
Discount rate	9	Report the choice of discount rate(s) used for costs and outcomes and say why appropriate.	N/A
Choice of health outcomes	10	Describe what outcomes were used as the measure(s) of benefit in the evaluation and their relevance for the type of analysis performed.	p.5-6/ 194-201
Measurement of effectiveness	11a	*Single study-based estimates:* Describe fully the design features of the single effectiveness study and why the single study was a sufficient source of clinical effectiveness data.	p.6-8/ 213-269

For peer review only

	11b	*Synthesis-based estimates:* Describe fully the methods used for identification of included studies and synthesis of clinical effectiveness data.	N/A
Measurement and valuation of preference based outcomes	12	If applicable, describe the population and methods used to elicit preferences for outcomes.	N/A
Estimating resources and costs	13a	*Single study-based economic evaluation:* Describe approaches used to estimate resource use associated with the alternative interventions. Describe primary or secondary research methods for valuing each resource item in terms of its unit cost. Describe any adjustments made to approximate to opportunity costs.	p.5-6/ 194-201
	13b	*Model-based economic evaluation:* Describe approaches and data sources used to estimate resource use associated with model health states. Describe primary or secondary research methods for valuing each resource item in terms of its unit cost. Describe any adjustments made to approximate to opportunity costs.	p.4-5/ 146-201
Currency, price date, and conversion	14	Report the dates of the estimated resource quantities and unit costs. Describe methods for adjusting estimated unit costs to the year of reported costs if necessary. Describe methods for converting costs into a common currency base and the exchange rate.	N/A
Choice of model	15	Describe and give reasons for the specific type of decision-analytical model used. Providing a figure to show model structure is strongly recommended.	p.7/ 243-206
Assumptions	16	Describe all structural or other assumptions underpinning the decision-analytical model.	N/A
Analytical methods	17	Describe all analytical methods supporting the evaluation. This could include methods for dealing with skewed, missing, or censored data; extrapolation methods; methods for pooling data; approaches to validate or make adjustments (such as half cycle corrections) to a model; and methods for handling population heterogeneity and uncertainty.	N/A
**Results**			p.6-8/ 214-269
Study parameters	18	Report the values, ranges, references, and, if used, probability distributions for all parameters. Report reasons or sources for distributions used to represent uncertainty where appropriate. Providing a table to show the input values is strongly recommended.	N/A
Incremental costs and outcomes	19	For each intervention, report mean values for the main categories of estimated costs and outcomes of interest, as well as mean differences between the comparator groups. If applicable, report incremental cost-effectiveness ratios.	p 8-13/ 272-348
Characterising uncertainty	20a	*Single study-based economic evaluation:* Describe the effects of sampling uncertainty for the estimated incremental cost and incremental effectiveness parameters, together with the impact	N/A

For peer review only

		of methodological assumptions (such as discount rate, study perspective).	N/A
	20b	*Model-based economic evaluation:* Describe the effects on the results of uncertainty for all input parameters, and uncertainty related to the structure of the model and assumptions.	N/A
Characterising heterogeneity	21	If applicable, report differences in costs, outcomes, or cost-effectiveness that can be explained by variations between subgroups of patients with different baseline characteristics or other observed variability in effects that are not reducible by more information.	p.8-13/ 272-348
**Discussion**			
Study findings, limitations, generalisability, and current knowledge	22	Summarise key study findings and describe how they support the conclusions reached. Discuss limitations and the generalisability of the findings and how the findings fit with current knowledge.	p.13-17/ 350-495
**Other**			
Source of funding	23	Describe how the study was funded and the role of the funder in the identification, design, conduct, and reporting of the analysis. Describe other non-monetary sources of support.	p.17/ 498-501
Conflicts of interest	24	Describe any potential for conflict of interest of study contributors in accordance with journal policy. In the absence of a journal policy, we recommend authors comply with International Committee of Medical Journal Editors recommendations.	p.18/ 530

For consistency, the CHEERS Statement checklist format is based on the format of the CONSORT statement checklist

The **ISPOR CHEERS Task Force Report** provides examples and further discussion of the 24-ite[illegible] CHEERS Checklist and the CHEERS Statement. It may be accessed via the *Value in Health* link [illegible] ISPOR Health Economic Evaluation Publication Guidelines – CHEERS: Good Reporting Practice[illegible] webpage: http://www.ispor.org/TaskForces/EconomicPubGuidelines.asp

The citation for the CHEERS Task Force Report is:

Husereau D, Drummond M, Petrou S, et al. Consolidated health economic evaluation reporting standards (CHEERS)—Explanation and elaboration: A report of the ISPOR health economic evaluations publication guidelines good reporting practices task force. Value Health 2013;16:231-50.

For peer review only

BMJ Open

Patient Level Cost of Diabetes Self-Management Education Programmes: An International Evaluation

Journal:	*BMJ Open*
Manuscript ID	bmjopen-2016-013805.R2
Article Type:	Research
Date Submitted by the Author:	01-Feb-2017

Complete List of Authors:	Doyle, Gerardine; University College Dublin, College of Business<br>O'Donnell, Shane; University College Dublin<br>Quigley, Etáin; University College Dublin<br>Cullen, Kate; University College Dublin<br>Gibney, Sarah; University College Dublin<br>Levin-Zamir, D ; University of Haifa,<br>Ganahl, Kristin; Gesundheit Osterreich GmbH<br>Mueller, Gabriele; Technische Universitat Dresden<br>Muller, Ingrid; University of Southampton, Department of Psychology<br>Maindal, Helle; Aarhus University<br>Chang, Wushou; School of Public Health, Taipei Medical University; Department of Occupational Medicine, Shuang Ho and Taipei Medical University Hospital<br>Van Den Broucke, Stephan; Universite catholique de Louvain, Psychological sciences Research Institute

**Primary Subject Heading**:	Health economics
Secondary Subject Heading:	Diabetes and endocrinology, Health policy, Health services research
Keywords:	Time Driven Activity Based Costing, Health literacy, Cost, Diabetes Mellitus, Type 2, Self-Management

SCHOLARONE™
Manuscripts

Patient Level Cost of Diabetes Self-Management Education Programmes: An International Evaluation

Author and Co-author's names:

Doyle, G., O'Donnell, S., Quigley, E., Cullen, K., Gibney, S., Ganahl, G., Müller, G, Muller, I., Maindal, H.T,. Chang. P,. Van den Broucke,. S.

The name, postal address, e-mail, telephone, and fax numbers of the corresponding author.

Corresponding Author:
Professor Gerardine Doyle:

University College Dublin
UCD College of Business
Q210, Lochlainn Quinn School of Business
Belfield
Dublin 4
Ireland
gerardine.doyle@ucd.ie
Tel: +353 1 716 4717
Fax: +353 1 716 4767

The full names, institutions, city, and country of all co-authors.

Names	Institutions	City	Country
Doyle, Gerardine	University College Dublin	Dublin	Ireland
O'Donnell, Shane	University College Dublin	Dublin	Ireland
Quigley, Etáin	University College Dublin	Dublin	Ireland
Cullen, Kate	University College Dublin	Dublin	Ireland
Gibney, Sarah	University College Dublin	Dublin	Ireland
Levin-Zamir, Diane	Clalit Health Services	Tel-Aviv	Israel
Ganahl, Kristin	Gesundheit Österreich GmbH	Vienna	Austria
Müller, Gabrielle	Technische Universität Dresden	Dresden	Germany
Muller, Ingrid	University of Southampton	Southampton	United Kingdom
Maindal, Helle Terkildsen	Aarhus University	Aarhus	Denmark
Chang, Peter	Taipei Medical University	Taipei	Taiwan
Van den Broucke, Stephan	Université Catholique de Louvain	Louvain	Belgium

Key words: Diabetes Mellitus Type 2, Self-management, Cost, Time Driven Activity Based Costing, Health Literacy

Word Count - excluding title page, references, figures and tables = 4,427.

For peer review only

Abstract

Objectives: The objective of this study was to examine the value of Time Driven Activity Based Costing (TDABC) in understanding the process and costs of delivering Diabetes Self-Management Education programmes (DSME) in a multi-country comparative study.

Setting: Outpatient settings in five European countries (Austria, Denmark, Germany, Ireland, United Kingdom) and two countries outside Europe, Taiwan and Israel.

Participants: Providers of DSME programmes across participating countries (N=16) including health care professionals, administrators and patients taking part in DSME programmes.

Primary and secondary measures: Primary Measure: Time spent by providers in the delivery of DSME and resources consumed in order to compute programme costs. Secondary measures: self-report measures of behavioural self-management and diabetes disease/health related outcomes.

Results: We found significant variation in costs and the processes of how DSME programmes are provided across and within countries. Variations in costs were driven by a combination of price variances, mix of personnel skill and efficiency variances. Higher cost programmes were not found to have achieved better relative outcomes. The findings highlight the value of TDABC in calculating a patient level cost and potential of the methodology to identify process improvements in guiding the optimal allocation of scarce resources in diabetes care, in particluar for DSME that is often underfunded.

Conclusions: This study is the first to measure programme costs using estimates of the actual resources used to educate patients about managing their medical condition and is the first study to map such costs to self-reported behavioural and disease outcomes. The results of this study will inform clinicians, managers and policy makers seeking to enhance the delivery of DSME programmes. The findings highlight the benefits of adopting a TDABC approach to understanding the drivers of the cost of DSME programmes in a multi-country study to reveal opportunities to bend the cost curve for DSME.

Article Summary

Strengths and limitations of this study

- Time Driven Activity Based Costing (TDABC) has rarely been applied to care pathways within non-acute settings and as such offers a novel perspective on understanding the costs of providing chronic disease self-management education.
- This is the first multi-country study to compare the costs of DSME across a number of countries within the EU and Asia.
- Outcomes of programme participation were measured through self-reported changes, making it difficult to establish if any clinical improvement occurred. Future studies should combine TDABC analysis with clinical outcomes to further assess value in DSME.

For peer review only - http://bmjopen.bmj.com/site/about/guidelines.xhtml

1.0 Introduction

Type 2 diabetes mellitus is one of the major public health threats of the 21st century, currently affecting approximately 59.8 million people within Europe and 415 million worldwide [1]. Further, it has been reported that diabetes medical care accounts for a disproportionate allocation of health service resources across the western world [1]. A recent US study performed an analysis of the spending on personal and public health across 155 conditions across time (1996-2013) and found that spending on diabetes (alongside low back and neck pain) increased the most over this period ($64.4 billion). Furthermore the study found that diabetes had the highest health care spending in 2013 ($101.4 billion), a disease attributable to behavioural or metabolic risk factors including diet, obesity, high fasting plasma glucose, tobacco use and low physical activity. [2] Developing the self-care capacity of patients is critical to determining optimal clinical, behavioural and psychosocial outcomes and therefore reducing costs[3]. Diabetes self-management education (DSME) has been shown to improve patient outcomes by reducing the onset and/or advancement of diabetes related complications; by improving quality of life; strengthening self-efficacy and personal empowerment; assisting with the development of healthy coping skills; and by reducing diabetes related distress and depression [4].

The operation and delivery of DSME varies across the international landscape. They can be either professionally led or peer led. Further, they can be group based, individually based, and increasingly IT based. In addition, DSME curricula, duration and delivery may vary substantially, both within and between countries[5]. It is well established that DSME programmes are a low cost intervention per patient and cost effective from a payer perspective. For example, a recent report published by *The Center For Health Law And Policy Innovation* (Harvard Law School) argues that cost savings can be made by public and private insurers in the United States if cost sharing were eliminated and DSME were provided free of charge to patients [6]. However, little research has explored why the costs of running such interventions vary across different health care systems and jurisdictions, or why these costs may differ. This study addresses this gap in the prior literature.

Indeed most of the economic analyses has thus far focused on establishing the cost effectiveness of DSME by comparing the cost of programmes relative to their clinical effectiveness. Such evaluations are usually based on economic modelling, carried out alongside randomised control trials and the findings typically suggest that DSME interventions are cost effective relative to usual care [7-113]. Despite this, a recent report published by the *Health Information and Quality Authority* (HIQA) [14] in Ireland highlights the large degree of heterogeneity in the methodological approaches used in such economic evaluations. This, in turn, makes results difficult to compare in any meaningful way. In

For peer review only

addition, these approaches tend to focus solely on overall cost of running the programmes and neglect to explore potential mechanisms through which DSME programmes could be made more efficient whilst also maintaining high standards of effectiveness. Furthermore, the majority of studies are based on interventions within a US population, and as such may not be generalizable across differing health care, social and cultural contexts.

This study seeks to address these existing gaps in the literature through an economic evaluation of DSME delivery across a number of EU and non-EU countries, namely Austria, Denmark, Germany, Ireland, Israel, Taiwan and the UK. The selection of these countries was based on access of the Diabetes Literacy Consortium[i] to local knowledge and networks required to carry out the necessary fieldwork. These countries also represent a diversity of contrasting approaches to the delivery of DSME tailored to each country [5]. The findings are part of a wider study conducted by the Diabetes Literacy Consortium, the overall purpose of which was to examine the (cost)-effectiveness of diabetes education across Europe, Israel, Taiwan and the US[ii]. The objective of this study is to examine the value of Time Driven Activity Based Costing (TDABC) in understanding the process and costs of delivering Diabetes Self-Management Education programmes (DSME) in multiple countries and sites (7 countries, 16 sites) and to identify potential process improvements in the delivery of such programmes to reveal opportunities to bend the cost curve for DSME.

2.0 Method

A Time Driven Activity Based Costing (TDABC) method was used to map the process of programme delivery and to derive patient level costs[15-16]. TDABC has been developed as a viable costing method for the health care sector by Kaplan and Porter [17-18] enabling detailed patient level costs to be computed alongside the identification of possible process improvements resulting in potential cost savings. TDABC is particularly compatible with type 2 diabetes care as the model can be applied to diverse care pathways, particularly chronic disease management. Adopting a TDABC approach in this study therefore gave increased visibility into areas of DSME delivery where process improvements and cost savings could be made, while still maintaining a high quality of patient education. Examples of the application of TDABC have been mostly confined to medical conditions and to acute clinical settings[18-20]. This study seeks to add to this body of knowledge on the costs of care within outpatient environments through identifying the patient level cost of a variety of DSME programmes both

---

[i] The Diabetes Literacy Consortium represents a group of countries funded by the European Commission under the Seventh Framework research programme (Grant Agreement Number: 306186).
[ii] http://www.diabetesliteracy.eu

For peer review only - http://bmjopen.bmj.com/site/about/guidelines.xhtml

cross-nationally and *Intra*nationally[21]. A primary objective was to provide a robust costing framework within which future studies could include clinical and quality of life outcomes to determine the economic value added to diabetes care through the use of DSME.

The TDABC method involves seven steps [17] 1) select the medical condition and/or patient population to be examined; 2) define the care value chain; 3) develop process maps of each activity in patient care delivery; identify the resources involved and any supplies used for the patient at each process step; 4) obtain time estimates for each process step; 5) estimate the cost of supplying each patient care resource; 6) estimate the practical capacity of each resource provided and calculate the capacity cost rate; 7) compute the total costs over each patient's cycle of care. By constructing a sequential activity and process step map and care value chain the researcher can analyse the maps/care pathway for duplication. These areas can then be explored further to establish if changes in the pathway would add value by maintaining/increasing the level of care to the patient whilst decreasing the economic cost to the overall health care system in terms of providing DSME programmes.

Each international study team identified the care value pathway in their country and collected the activity and time data related to the care value pathway, through qualitative semi-structured interviews of health care providers from each education programme (N=16). These included physicians, nurses, educators and managers. This information was then entered into an aggregated, de-identified database for analysis. All study teams then collected resource and financial data, utilising a standardised costing worksheet related to the activities, which were then incorporated into the aggregated database for analysis. This methodology was applied to each education programme across each country included in the study. The topic guide was developed in the English language and was then subsequently translated into the local language by the local research teams in each of the participating countries.

All activities associated with the DSME pathway were entered into an aggregated Excel database. All activity and time data was collected via the survey instrument, and cost estimates were assigned to these activity variables using financial data provided by the local provider organizations.

DSME programme costs were derived specifically from the cost of performing each activity in the delivery of the programme. All cost data was entered into activity spread-sheets and therefore the data collected did not contain any information relating to identifiable individual service providers. In

For peer review only - http://bmjopen.bmj.com/site/about/guidelines.xhtml

the resulting database, all cost information was linked to activities and not to individuals. All activity and cost information is reported per DSME programme.

To compare the outcomes of the DSME programmes, a multi-centre observational pre-post study design was used involving diabetes patients enrolled in each of the DSME programmes. Data from the participants were collected at the beginning of the programme and after three to six months. The programmes included in the study were existing programmes using five different modes of delivery: individual education in one-on-one sessions, beyond routine treatment provided, group education, self-help groups, or a combination of some of the above delivery modes. The content of peer-led and structured DSME programmes was not comparable. Therefore the two peer-led programmes were excluded from our data analysis.

2.1 Study Sample

To be selected for inclusion, programmes had to: (1) target type 2 diabetes patients; (2) be conducted among the general patient population rather than tailored to the needs of a specific age cohort, needs or gender group; (3) include (but not be limited to) newly diagnosed patients; (4) be stand-alone programmes rather than an add-on to another programme or part of a wider curriculum with (multiple) parallel programs; (5) admit new patients during the time of the baseline data collection. The study sample size was driven by the number of programmes involved in the delivery of the specific DSME programmes in each country. Costs were collected for the duration of each programme, which ranged in duration from one day to those spanning a 12-month timeframe.

2.2 Analytic approach

The Time Driven Activity Based Costing (TDABC) model was utilised to calculate a cost per programme. Significant variations in programme costs prevailed despite broadly similar programme curricula across countries and programmes. Data collected revealed significant variation in number of education hours across the programmes, number and types of personnel delivering the programmes, practitioner hours and number of participating patients.

Two concepts and measures were drawn upon to develop the TDABC model[17], the unit cost of supplying capacity and the time it takes to undertake an activity. First, the model was used to calculate the cost of all the resources supplied to each programme. This included personnel, supervision and overheads including rent, equipment and software and insurance. The total cost was then divided by the actual capacity in order to calculate the cost rate. Second, the capacity cost rate was used to assign the programme costs to objects by estimating demand on the resource. Two

For peer review only - http://bmjopen.bmj.com/site/about/guidelines.xhtml

variables were estimated: the capacity cost rate for the programme and the capacity use by each patient. The capacity cost rate was calculated by:

$$\text{Capacity Cost Rate} = \frac{\text{Cost of Capacity Supplied}}{\text{Practical Capacity of Resource Supplied}}$$

Practical capacity was used as the denominator in the capacity cost rate equation. Estimating the practical capacity required two time estimates which were gathered from Human Resources and other administrative records: the total number of days that each employee actually worked each year; the total number of hours per day that the employee was available for work. Practical capacity was calculated as 80% of this working time [17]. Therefore 20% was attributed to breaks, training and annual leave. This was applied to all countries to ensure consistency and comparability of the computed programme costs.

In order to calculate the total cost of each DSME programme per patient, the capacity cost rates (including associated support costs) for each resource used was multiplied by the amount of time attributed to each patient. This calculation was based on the number of patients enrolled at the outset of the programme. The total cost of each programme per patient was the sum of all the costs across all the processes within the DSME programme. The costs were collected in the local currency and then expressed in international dollar to ensure comparability of the costs by using the Purchasing Power Parity conversion factors, to control for different standards of living, different wage levels across countries and for the particular exchange rate.

As suggested by Erhun et al. we performed a quantitative investigation of the differences in consumption and pricing of labour resoucres using cost variance analysis on labour costs. This analysis enabled us to quantitatively discern differences between processes at selected sites. The cost difference can be divided into two effects, a price variance (due to different capacity cost rates of labour resource (CCR)) and a quantity variance (due to different use of the labour resource across the sites). We performed this variance analysis to understand the differences in consumption and pricing of labour resources and to understand the drivers of cost variation across capacity cost rate variances, mix of personnel and efficiency variances[18]

To understand the association between programme cost and health outcomes achieved, we mapped the cost per programme to self-reported patient outcomes. Due to the significant difference in

For peer review only - http://bmjopen.bmj.com/site/about/guidelines.xhtml

access to clinical data across the participating countries in this study, it was not possible to collect comparable clinical data for each country. Therefore comparable data was collected to measure outcomes at behavioral and disease/health outcome levels for existing diabetes self-management programs. Health outcome data was collected at three levels; individual diabetes self-management disposition, behaviour and disease/health related outcomes. (The outcome framework employed in this study is summarised in the Supplemental File attached.)

3.0 Results

Findings highlight that the programmes included in this study provide similar educational content when delivering diabetes education. Further, we found similar changes in self-reported behavioural and disease outcomes across programmes. This suggests that factors other than educational content drives cost variation across programmes and despite reported cost variation, outcomes appear broadly similar. The cost difference between two sites can be analysed into two effects: a price variance due to different capacity cost rates of resource and a quantity variance due to different use of resource:

$$\Delta_{1,2} = \sum_{i=1}^{N_L} q_1^i \times r_1^i - \sum_{i=1}^{N_L} q_2^i \times r_2^i$$

Figure 1 presents the price variance across the sites[iii].

Insert Figure 1 here.

There are a number of factors which were found to influence cost variation. Firstly, programmes differed in duration and hours of practitioner time spent on each programme delivery. This reflects the efficiency variance due to different quantities of total personnel used. For example, Figure 2 highlights that the 'Ireland 2' programme utilises 78 hours of personnel, whereas 'Austria 3' only uses 5.25 hours of personnel time, yet patient self-reported outcomes are broadly similar. This suggests that total personnel time is a strong cost driver but not an outcome driver. This efficiceny variance across two sites is expressed as:

[iii] For each Figure 1-6 the associated data is included in Supplemental Files attached.

For peer review only - http://bmjopen.bmj.com/site/about/guidelines.xhtml

$$= \left(\sum_{i=1}^{N_L} r_2^i \times \frac{q_2^1}{Q_2} \right) \times (Q_1 - Q_2)$$

Insert Figure 2 here.

Secondly, mix of personnel skill used in providing the education is a cost driver. For example, the high salary cost for a consultant physician in Germany and social worker cost in Israel (Figure 3) did not produce any significant improvement in patient self-reported outcomes. These findings suggest that personnel skill used is a strong cost driver but does not significantly alter patient self-reported outcomes. When comparing two sites this mix variance is measured as follows:

$$= \left(\sum_{i=1}^{N_L} \left(\frac{q_1^i}{Q_1} - \frac{q_2^i}{Q_2} \right) \times r_2^i \right) \times Q_1$$

Insert Figure 3 here.

Figure 3 presents the weighted average capacity cost rate, the weights representing the percentage of total time used of each personnel type. This highlights both the variety of personnel type used across DSME programmes and countries in addition to the differing percentage of total time used of each personnel type.

Thirdly, the number of patients who attended each programme was a strong per-patient cost driver (Figure 4), generally the more patients who attended the programme the lower the per-patient cost.

Insert Figure 4 here.

Taking total cost per programme, the median programme was identified as Austria Programme 1. The key cost drivers identified were then compared to this base programme to explore the behaviour of these variances. Figure 5 summarises this comparison with the base country and reveals that there is a non-linear relationship between the cost of a programme (dependent variable) and each of the key cost drivers (independent variables); practitioner hours used, the

For peer review only - http://bmjopen.bmj.com/site/about/guidelines.xhtml

weighted average capacity cost rate and the number of patients participating on the programmes. In general for practitioner hours, weighted average CCR and patient numbers, as the price variance from the median increases, so too do of these independent variables. However there are some exceptions to this general trend; UK 2 and Ireland 1 where a lower number of practitioner hours are used, Israel 2 and UK 2 where a lower weighted average CCR can be observed and Ireland 2 and UK 2 where there is a lower number of participating patients.

All programmes with a lower cost than the median do have lower practitioner hours, lower weighted average CCR and a lower number of patients, but not proportionately lower. Programme UK 2 appears to be the programme which has a cost higher than the median and yet consistently has a lower number of practitioner hours, lower weighted average CCR and lower patient numbers than the median. This reveals the complexity of the cost behaviours and of the cost variations between the programmes despite offering similar curricula and resulting in similar health outcomes.

Insert Figure 5 here.

There was very little variation in outcomes across each of the programmes, both within and between countries, whatever the mode of delivery, mix of personnel skill used, quantity of total personnel hours, quantity of education hours or quantity of participating patients. For simplicity, Figure 6 maps the health outcomes of one particular variable only, general diet, alongside the cost per programme (Figures 6a and 6b). Very modest improvements in general diet were achieved after participation in DMSE and higher cost programmes did not result in better health outcomes. For example, Israel 2 programme recorded the largest change in health outcomes at a low cost in comparison with the most expensive programme, Ireland 2, which resulted in a very small change in health outcomes. Although only general diet is illustrated here, other outcome data show that DSME was only weakly helpful or in some cases had no effect at all on the health outcomes of participants. (Supplemental File Outcomes Framework and Outcomes Data: Table 2: Self-reported Patient Outcomes.)

Insert Figures 6a and 6b here.

4.0 Discussion

The data illustrates that Diabetes Self Management Education programmes are provided at a low cost in every country studied. The data provides evidence that while these costs are low, significant

For peer review only - http://bmjopen.bmj.com/site/about/guidelines.xhtml

cost variations exist both within and between countries. This is due to a combination of cost variations between the programmes; the capacity cost rate, the mix of personnel delivering the education, the different quantities of total personnel used and the number of patients participating in these programmes. This is the first time that such multi-country comparative data has been collected.

The variance analysis performed surrounding costs and outcomes illustrates total personnel hours as a strong cost driver (Figure 2). Practitioners such as nurses and diabetes nurse specialists can produce similar outcomes to physicians but at a lower salary and practical capacity cost. This is likely to be a more effective use of resources, particularly in relation to optimizing use of personnel at their level of expertise. Further research is needed to explore the most appropriate level of expertise required to deliver the programme for optimal patient health outcomes. For example, instead of having a consultant physician or a Clinical Nurse Specialist delivering the education programmes it may be more appropriate to have well trained experienced nurses or the equivalent performing this role. A pilot study conducted by Kaplan et al. at The University of Texas Cancer Centre revealed that matching clinical skills to the processes led to a 16% reduction in process time, a 12% decrease in costs for technical staff, and a 67% reduction in costs for professional staff[17]. However clinical outcomes, in addition to behavioural and psychosocial outcomes, are necessary to determine fully whether the educators' level of expertise really influences all DSME health outcomes[17].

In some countries the cost of the same programme varied by site. For these programmes, we observed significant variation in administrative hours and this was not associated with the number of participating patients. This finding is similar to that of Munoz et al. who used TDABC in a cost-effectiveness analysis of a red blood cell salvage post total-knee arthroscopy in the US, Switzerland and Austria and suggest that tighter control of administrative costs may reduce what appear to be non-value added hours for the patients[22].

Integrating data on the number of patients participating on each programme (Figure 4) with the outcome data suggests that the number of patients in attendance did not impact on patient self-reported outcomes. These findings suggest that there is room for cost savings in DSME regarding the amount of hours of education provided, who provides the education and the number of patients in attendance at each programme, without negatively impacting patients self-reported outcomes.

A number of learnings emerged from this study: firstly, all programme curricula covered similar topics, this suggests that there is a shared consensus on what information requires dissemination

For peer review only - http://bmjopen.bmj.com/site/about/guidelines.xhtml

and highlights that variation relates to process delivery rather than curricula; secondly, the administrative burden on programmes varies greatly and as such is an area of programme development which requires planning and streamlining; thirdly, the skill mix of professionals delivering the programme varies greatly suggesting a lack of empirical knowledge surrounding the most effective educator; fourthly, the duration and hours of education varies significantly across sites, again highlighting a lack of consensus in terms of the most efficacious course construct; and finally, such cost variation exists across sites despite the programme content being broadly similar. The granular mapping of the DSME programmes and the derivation of a cost per programme is the first step in generating a better understanding of the DSME arena internationally.

A separate analysis of the self-reported outcome data was conducted by Peer et al. analysing the DSME data for all programmes in aggregate[23]. They found that these outcomes were similar irrespective of the education programme or the country (albeit that the sample size was small and the standard deviation high). When the programmes were analysed in aggregate, a statistically significant improvement was found for six behavioral outcomes (general diet, exercise, medication use, problem areas in diabetes, foot care and appraisal of diabetes) and three disease/health related outcomes (BMI, health related quality of life and affective well-being). (Please see the Supplemental File Outcomes Framework and Outcomes Data Table 3 and related note explaining the precise scales used.)

The costing and provision of DSME is at an early stage of development globally with limited empirical knowledge of the most efficient and effective mode of delivering DSME. Thus this study has gone some way to remedying this problem whereby it has outlined a bottom up/patient level cost using estimates of the actual resource costs used to educate patients through self-management programmes and therefore a more accurate cost than heretofore of providing various education programmes. Thus, it has provided a first layer of information, which in the future will be required to establish whether this model of care/intervention can add value to the health care system once clinical effectiveness outcomes have been determined for each programme. Storfjell et al., show that the application of TDABC in the context of nursing care can facilitate the identification and elimination of non-value added time (NVA) and related the increase in time spent on psychosocial intervention, support and patient education[24]. However, there is a long way to go, whereby clinical and Quality of Life outcomes are required to measure the effectiveness of DSME programmes before a thorough understanding of their added value to patients can be estimated. The methods and results of the current study will inform future research to achieve a better understanding of the

For peer review only - http://bmjopen.bmj.com/site/about/guidelines.xhtml

added value derived from providing DSME interventions. We suggest that future studies include a rigorous collection of clinical outcomes pre and post DSME.

5.0 Limitations

The TDABC method is a relatively new method in terms of healthcare costing and to the best of the authors' knowledge has yet to been applied to investigate the costs of a health education intervention. As a result there were limited guidelines surrounding the collection of activity and process step data in non-acute settings and thus it was necessary for the research team to develop such a protocol that was fit-for-purpose across different international study sites. In practice, many participants were unfamiliar with the costing and activity terminology and the level of detail required on all forms of activity, for TDABC. We observed that participants appeared to provide less detail on administrative and programme preparation activity compared with education activity. This detailed information would have provided greater insight into the reasons why administrative costs were found to be so high in some countries while not in others.

In addition, some of the local research teams also experienced difficulties in collecting the required financial data. For example, in Belgium, the staff involved in the delivery of DSME programmes taking part in this study, were unable to disclose personal salary information, which was not otherwise available from a public source, as in other countries. This related to data protection legislation (enacted 1992, subsequently amended 1998, 2003), together with the fact that there is no professional category of diabetes educator in Belgium. For these reasons, the Belgian data had to be excluded from this particular study.

The study is also limited by a lack of available clinical outcome data from each of the education programmes. While important self-reported health and psycho-social outcome data was collected in each country it was not possible to determine the clinical-effectiveness of these DSME programmes in terms of glycemic control due to the absence of any clinical measures. As Kaplan and Porter point out[17], value in health care can only be determined when there is visibility into both costs and *clinical* outcomes. Furthermore, the reliability of self-reported outcomes data was undermined by small sample sizes in each country. Secondly, self-reported measures of health behavior are susceptible to social desirability bias, and response styles can vary by culture and setting [25] [26] Nonetheless, the similarity in outcomes across each of the sites regardless of the amount of money invested in each programme raises questions surrounding the value being achieved per Euro/ dollar spent.

For peer review only - http://bmjopen.bmj.com/site/about/guidelines.xhtml

The peer-led programmes found in Denmark and Germany were excluded from the analysis. However, they were provided at the lowest cost of Int $0.15 and Int $0.74 per patient per hour of education respectively. When self-assessed outcome data was measured for each programme, the outcomes were similar for peer-led and specialist-led programmes. We suggest that further research is needed surrounding peer-led education and measurement of associated clinical health outcomes.

6.0 Conclusion

This paper has demonstrated the variances in the cost of delivering different types of diabetes education programmes, both within and across countries in the EU and Asia. Developing cost effective lifestyle interventions to improve the diabetes knowledge and self-management skills and quality of life for patients may be an important step in preventing the onset of complications associated with type 2 diabetes. The imperative to do so from an economic perspective cannot be underestimated when consideration is given to the implications for health care systems associated with the treatment of diabetes related morbidities such as active foot disease, chronic kidney disease, retinopathy and myocardial infarction[27].

This study offers the first application of a TDABC approach to evaluate the cost of delivering DSME programmes and as a means of comparing the costs of running a healthcare intervention cross-nationally. It contributes to the extant literature by highlighting and describing the vast combinations and permutations of delivery of DSME curricula, practitioner hours, hours of education, mix of educators, numbers of attendees and how these variations lead to substantial cost differences. Our variance analysis revealed that the key drivers of cost variation arose from differing weighted average capacity cost rates representing the percentage of total time used of each personnel type, the mix of personnel delivering the education and the number of patients participating in these programmes. In the process, we identified how there could be potentially unnecessary process steps that, if eliminated, could lead to cost savings in the delivery of DSME programmes, including vast differences in administration time, and exploring the mix of personnel skill alongside the total personnel time used.

While it is already established that diabetes education is a low cost intervention and is cost-effective, given the sheer numbers of education programmes that need to be made available to meet the demands resulting from increasing levels of diabetes worldwide, even small process improvements could lead to overall cost savings for healthcare providers. Future studies focusing on the cost-

For peer review only - http://bmjopen.bmj.com/site/about/guidelines.xhtml

effectiveness of healthcare interventions may consider adopting TDABC principles and variance analysis as a means of identifying efficiencies in other chronic disease education programmes.

The study has highlighted the strengths of TDABC as a method of bottom up costing in outpatient care and recommends utilising this method in future studies so as to allow for a comprehensive literature to develop in the area, enabling comparative studies to be performed. By developing such literature a comprehensive understanding of the cost of patient education programmes can be developed and compared cross nationally and across time. Health care practitioners and educators who wish to convince policy makers and health insurers to reimburse the cost of DSME delivery can adopt a TDABC approach in order to demonstrate that such programmes are run efficiently and effectively especially when combined with measures of consequent clinical health outcomes to represent value for money.

Acknowledgements

This article is part of the Diabetes Literacy project supported by grant FP7-Health-2012-Innovation-1/306186 of the European Commission. The authors would like to thank the partners of the Diabetes Literacy Consortium for contributing to data collection with participants and providers of DSME programs in their respective countries.

The Diabetes Literacy Consortium consists of the following members:

Université Catholique de Louvain, Belgium: Stephan Van den Broucke, Gerard Van der Zanden, Marie Housiaux, Louise Schinckus.

Technische Universität Dresden, Medical Faculty, Germany: Peter Schwarz, Gabriele Mueller, Henna Riemenschneider, Sarama Saha.

University College Dublin, Ireland: Gerardine Doyle, Shane O'Donnell, Etain Quigley, Kate Cullen, Sarah Gibney

Gesundheit Österreich GmbH, Austria: Jürgen Pelikan, Florian Röthlin, Kristin Ganahl, Sandra Peer.

Maastricht University, Department of International Health, The Netherlands: Helmut Brand, Kristine Sörensen, Timo Clemens, Marjo Campmans

University of Southampton, UK: Lucy Yardley, Ali Rowsell, Ingrid Muller, Victoria Hayter

Clalit Health Services, Israel: Diane Levin-Zamir, Ziv Har-Gil.

University of California at San Francisco, USA: Dean Schillinger, Courtney Lyles, Lina Tieu.

For peer review only - http://bmjopen.bmj.com/site/about/guidelines.xhtml

For peer review only

Taipei Medical University, Taiwan: Peter Chang, Candy Kuo, Alice Lin, Duong Van Tuyen, Becky Sun.

Aarhus University, Denmark*:* Helle Terkildsen Maindal, Jill Rowlands, Ulrik Martensen.

We also thank all the medical and educational services from the eight participating countries who contributed to the data collection.

Contributorship statement GD initially proposed the study. GD and SG specified the methodology. KC carried out the cost analysis. All authors contributed to the protocol design, data collection and analysis plan. EQ and SO'D wrote the initial manuscript, and all authors contributed to improving the manuscript. All authors approved the final manuscript.

Competing interests None declared.

Ethics Approval All methods were approved by the SVUH group Research and Ethics Committee, by the Research Ethics Committee of the Office of Research Ethics, University College Dublin and by the relevant local ethics committees in each jurisdiction and each study site where the study was carried out.

Funding This study is part of the Diabetes Literacy project supported by grant FP7-Health-2012-Innovation 1/306186 of the European Commission.

Data sharing statement The Excel spreadsheets showing how the individual activity costs were aggregated are available should they be requested.

References

1. International Diabetes Federation. IDF Diabetes Atlas. 7 ed ed. Brussels: International Diabetes Federation, 2015.
2. Dieleman, J.L., Baral, R., Birger, M., et al. US spending on personal health care and public health, 1996-2013. JAMA. 2016: 316(24):2627-2646. Doi:10.1001/jama.2016.16885.
3. Peyrot M, Burns KK, Davies M, et al. Diabetes Attitudes Wishes and Needs 2 (DAWN2): A multinational, multi-stakeholder study of psychosocial issues in diabetes and person-centred diabetes care. Diabetes Research and Clinical Practice 2013;**99**(2):174-84.
4. Powers MA, Bardsley J, Cypress M, et al. Diabetes Self-management Education and Support in Type 2 Diabetes A Joint Position Statement of the American Diabetes Association, the American Association of Diabetes Educators, and the Academy of Nutrition and Dietetics. The Diabetes Educator 2015;**41**(4):417-30.
5. Saha S, Müller G., Riemenschneider H, et al. Compendium of Diabetes Self-Management Education Programs in the European Union Member States, Israel, Taiwan and the USA. Version 1.1, Dresden, 2015.
6. Garfield K, Downer S, Rosenberg A, et al. Reconsidering Cost Sharing for DSME: Recommendation for Policy Reform. Massachusetts: Harvard, 2015.
7. Gillespie P, O'Shea E, Paul G, et al. Cost effectiveness of peer support for type 2 diabetes. International journal of technology assessment in health care 2012;**28**(01):3-11.

For peer review only - http://bmjopen.bmj.com/site/about/guidelines.xhtml

8. Brown 3rd H, Wilson KJ, Pagán JA, et al. Cost-effectiveness analysis of a community health worker intervention for low-income Hispanic adults with diabetes. Prev Chronic Dis 2012;**9**:E140.

9. Brownson CA, Hoerger TJ, Fisher EB, et al. Cost-effectiveness of Diabetes Self-management Programs in Community Primary Care Settings. The Diabetes Educator 2009;**35**(5):761-69.

10. Kuo S, Bryce CL, Zgibor JC, et al. Cost-Effectiveness of Implementing the Chronic Care Model for Diabetes Care in a Military Population. Journal of Diabetes Science and Technology 2011;**5**(3):501-13.

11. Jacobs-van der Bruggen MA, van Baal PH, Hoogenveen RT, et al. Cost-effectiveness of lifestyle modification in diabetic patients. Diabetes Care 2009;**32**(8):1453-58.

12. Dall TM, Roary M, Yang W, et al. Health care use and costs for participants in a diabetes disease management program, United States, 2007–2008. Prev Chronic Dis 2011;**8**(3):A53.

13. Molsted S, Tribler J, Poulsen PB, et al. The effects and costs of a group-based education programme for self-management of patients with Type 2 diabetes. A community-based study. Health Education Research 2012;**27**(5):804-13.

14. Health Information and Quality Authority. Health technology assessment of chronic disease self-management support interventions. Dublin: HIQA, 2015.

15. Kaplan R, Anderson SR. Time-driven activity-based costing: a simpler and more powerful path to higher profits. Harvard business press 2013

16. Kaplan RS, Anderson SR. The innovation of time-driven activity-based costing. Journal of cost management 2007;**21**(2):5-15.

17. Kaplan RS, Porter ME. How to solve the cost crisis in health care. Harvard Business Review 2011;**89**(9):46-52.

18. Erhun F, Mistry B, Platchek T, et al. Time-driven activity-based costing of multivessel coronary artery bypass grafting across national boundaries to identify improvement opportunities: study protocol. BMJ open 2015;**5**(8):e008765.

19. Akhavan S, Ward L, Bozic KJ. Time-driven Activity-based Costing More Accurately Reflects Costs in Arthroplasty Surgery. Clinical Orthopaedics and Related Research® 2016;**474**(1):8-15.

20. Kaplan AL, Agarwal N, Setlur NP, et al. Measuring the cost of care in benign prostatic hyperplasia using time-driven activity-based costing (TDABC). Healthcare 2015;**3**(1):43-48.

21. Demeere N, Stouthuysen K, Roodhooft F. Time-driven activity-based costing in an outpatient clinic environment: Development, relevance and managerial impact. Health Policy 2009;**92**(2–3):296-304.

22. Muñoz M, Campos A, Martín-Montañez E, et al. Could cost-effectiveness of postoperative shed blood salvage after elective knee arthroplasty be improved? Transfusion 2013;**53**(6):1372-73.

23. Peer,S. Ganahl, K., Roethlin, F., Baumann, L., Pelikan, J.M., Chang, P., Doyle, G., Juul, L., Levin-Zamir, D., Muller, I., Riemenschneider, H., Schillinger, D., Schinckus, L., Van den Broucke, Relative effectiveness of diabetes self-management education programs using different modes of delivery: Outcomes of Different Communication Channels of DSME programs. Presented at the 9th European Public Health Conference, Vienna, Austria, 9-12 November, 2016.

24. Storfjell JL, Ohlson S, Omoike O, et al. Non-Value-Added Time: The Million Dollar Nursing Opportunity. Journal of Nursing Administration 2009;**39**(1):38-45.

25. Herbert JR, Yunsheng M. Clemow L., Ockene IS. Saperia G. Stanek III EJ, Merriam PA, Ockene JS: Gender differences in social desirability and social approval bias in dietary self-report, *American Journal of Epidemiology*, 146:1046-1055, 1997.

26. Kristal A. Andrilla CH. Koepsell TD. Diehr PH.Cheadle A: Dietary assessment instruments are suspectible to intervention-associated resposne set bias, *Journal of the American Dietetic Association*, 98: 40-42, 1998.

27. Seuring T, Archangelidi O, Suhrcke M. The Economic Costs of Type 2 Diabetes: A Global Systematic Review. PharmacoEconomics 2015;**33**(8):811-31.

For peer review only - http://bmjopen.bmj.com/site/about/guidelines.xhtml

Figure Legend:

Figure 1: Cost Per Programme (Salary and Overheads) in International $

Figure 2: Percentage of total personnel time used per site

Figure 3: Weighted Average Capacity Cost Rate per Site

Figure 4: Number of Participants per Programme

Figure 5: Variance from Median Programme (Austria 1)

Figure 6a: Total Cost per Programme

Figure 6b: Change in Health Outcomes (General Diet) Following Participation across different DSME Sites

For peer review only

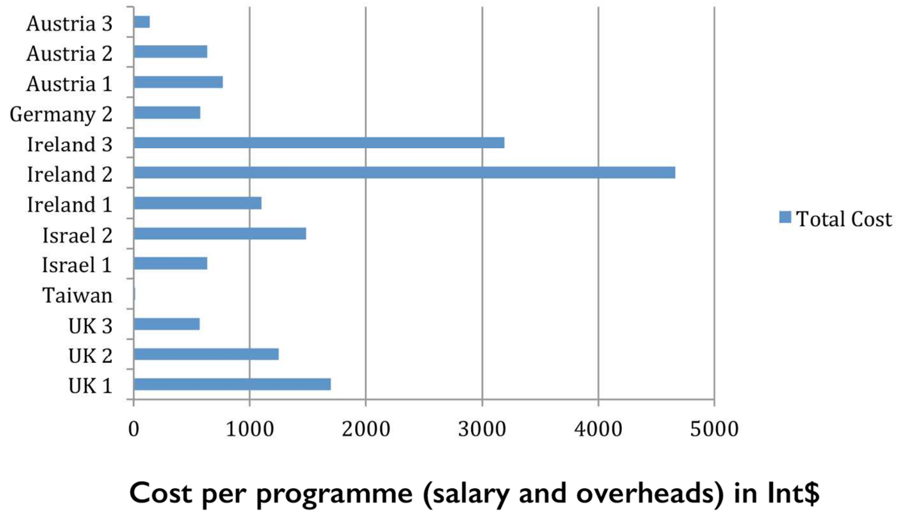

Cost per Programme (Salary and Overheads) in International $
Line 285
160x99mm (300 x 300 DPI)

For peer review only

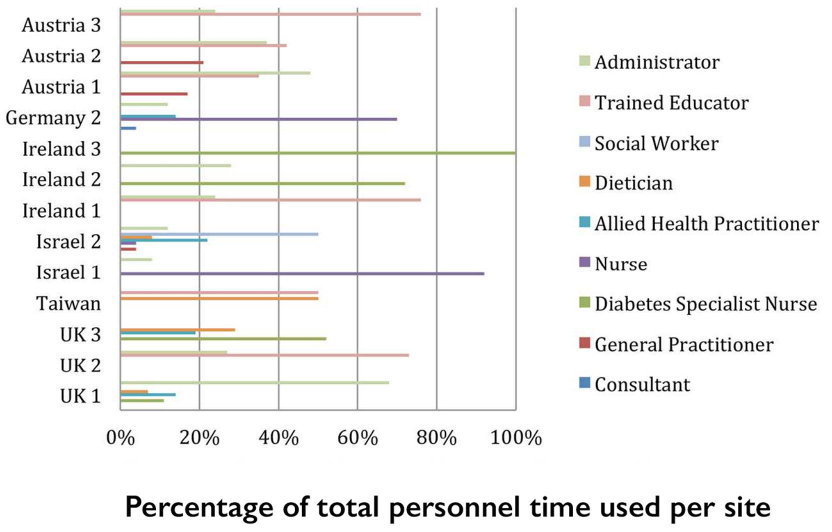

Percentage of Total Personnel Time Used per Site
Line 297
160x99mm (300 x 300 DPI)

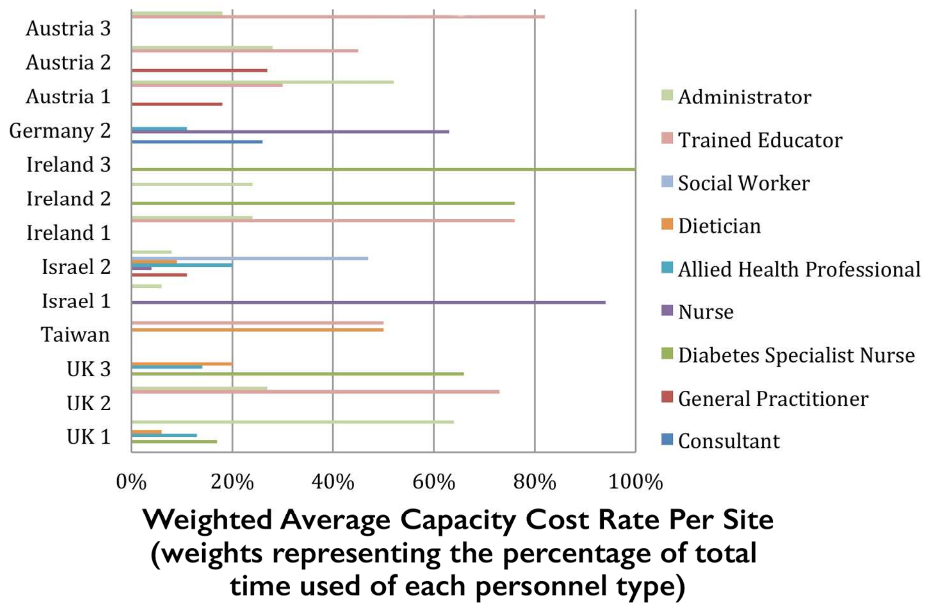

Weighted Average Capacity Cost Rate Per Site
Line 308
160x99mm (300 x 300 DPI)

For peer review only

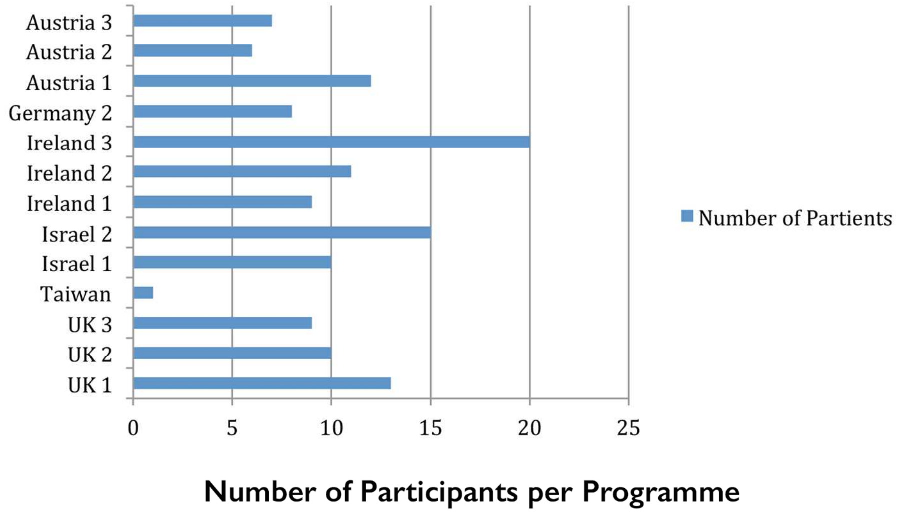

Number of Participants Per Programme
Line 319
160x99mm (300 x 300 DPI)

For peer review only

Programme	Total Cost	Price Variance from Median	Practitioner Hours	Prac Hrs Variance from Median	*Weighted Average CCR*	*Weighted Average CCR* Variance from Median	No. of Patients	No. of Patients Variance from Median
Ire 2	4664	3,898	78	55	3,825	3,022	11	-1
Ire 3	3193	2,427	61	38	2,515	1,712	20	8
UK 1	1697	931	57	34	1,560	757	13	1
Israel 2	1485	719	25	2	482	-321	15	3
UK 2	1248	482	13	-10	452	-351	10	-2
Ire 1	1099	333	19	-4	879	76	9	-3
Aus 1	766	0	23	0	803	0	12	0
Israel 1	633	-133	15	-8	346	-457	10	-2
Aus 2	633	-133	19	-4	536	-267	6	-6
Germany 2	573	-193	19	-4	417	-386	8	-4
UK 3	567	-199	12	-11	369	-434	9	-3
Aus 3	136	-630	5	-18	148	-655	7	-5
Taiwan	14	-752	5	-18	6	-797	1	-11

Variance from Median Programme (Austria 1)

Variance from Median Programme (Austria 1)
Line 339
160x99mm (300 x 300 DPI)

For peer review only

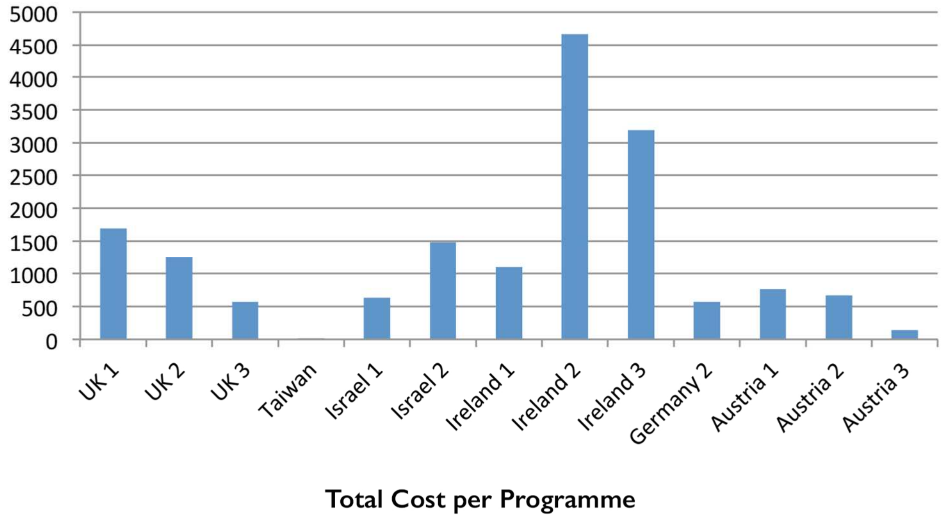

Total Cost Per Programme
Line 355
160x99mm (300 x 300 DPI)

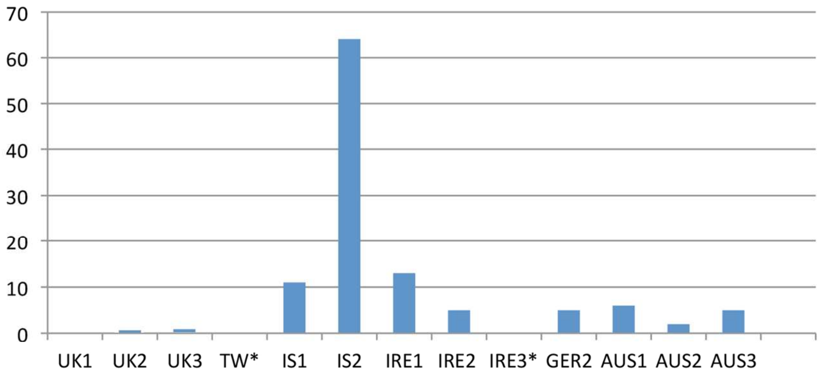

Change in Health Outcomes (General Diet)
following participation across different DSME sites

Change in Health Outcomes (General Diet) Following Participation across DSME Sites
Line 355
160x99mm (300 x 300 DPI)

review only

Data to Support Figures 1-5

**Programme**	Total Cost per-programme (home currency)	Total Cost per-programme (Int$)
UK 1	£1201.83	1697.50
UK 2	£883.60	1248.02
UK 3	£402.03	567.83
Taiwan	227.71 NTD	14.01
Israel 1	2532.47 ILS	633.11
Israel 2	5952.05 ILS	1484.52
Ireland 1	€923.38	1099.26
Ireland 2	€3918.31	4664.65
Ireland 3	€2682.40	3193.33
Germany 2	€451.52	573.72
Austria 1	€619.68	766.93
Austria 2	€536.28	663.71
Austria 3	€109.97	136.10

Figure 1 Data: Cost Per Programme (Salary and Overheads)

For peer review only

Cost Driver 1: Percentage of total personnel time used per site

**Staff (Hours) Programme**	Consultant	GP	DSN	Nurse	AHP	Dietician	Social Worker	Trained Educator	Administrator	Total time (Hours)
UK 1	-	-	6 (11%)	-	8 (14%)	4 (7%)	-	-	39 (68%)	57 (100%)
UK 2	-	-	-	-	-	-	-	15 (73%)	5.5 (27%)	20.5 (100%)
UK 3	-	-	5.5 (52%)	-	2 (19%)	3 (29%)	-	-	-	10.5 (100%)
Taiwan	-	-	-	-	-	2.4 (50%)	-	2.4 (50%)	-	4.8 (100%)
Israel 1	-	-	-	11.5 (92%)	-	-	-	-	1 (8%)	12.5 (100%)
Israel 2	-	.75 (4%)	-	.75 (4%)	4 (22%)	1.5 (8%)	9 (50%)	-	2 (12%)	18 (100%)
Ireland 1	-	-	-	-	-	-	-	14 (76%)	4.5 (24%)	18.5 (100%)
Ireland 2	-	-	56 (72%)	-	-	-	-	-	22 (28%)	78 (100%)
Ireland 3	-	-	60.6 (100%)	-	-	-	-	-	-	60.6 (100%)
Germany 2	.75 (4%)	-	-	13 (70%)	2.5 (14%)	-	-	-	2.25 (12%)	18.5 (100%)
Austria 1	-	4 (17%)	-	-	-	-	-	8 (35%)	11 (48%)	23 (100%)
Austria 2	-	4 (21%)	-	-	-	-	-	8 (42%)	7 (37%)	19 (100%)
Austria 3	-	-	-	-	-	-	-	4 (76%)	1.25 (24%)	5.25 (100%)

Figure 2 Data: Percentage of time for each personnel type per site

For peer review only

For peer review only - http://bmjopen.bmj.com/site/about/guidelines.xhtml

Cost Driver 2: Percentage of total salary cost (Int $) across personnel type and site

**Total staff cost per personnel type /prog.**	Consultant	GP	DSN	Nurse	AHP	Dietician	Social Worker	Trained Educator	Administrator	Total salary cost (Int $) per site
UK 1	-	-	264.60 (17%)	-	201.92 (13%)	100.96 (6%)	-	-	992.16 (64%)	1559.64 (100%)
UK 2	-	-	-	-	-	-	-	661.50 (73%)	121.55 (27%)	452.30 (100%)
UK 3	-	-	242.55 (66%)	-	50.48 (14%)	75.72 (20%)	-	-	-	368.75 (100%)
Taiwan	-	-	-	-	-	3.02 (50%)	-	3.02 (50%)	-	6.04 (100%)
Israel 1	-	-	-	325.60 (94%)	-	-	-	-	20.22 (6%)	345.82 (100%)
Israel 2	-	55.36 (11%)	-	21.23 (4%)	94.80 (20%)	42.47 (9%)	227.53 (47%)	-	40.45 (8%)	481.84 (100%)
Ireland 1	-	-	-	-	-	-	-	665.00 (76%)	213.75 (24%)	878.75 (100%)
Ireland 2	-	-	2897.44 (76%)	-	-	-	-	-	927.30 (24%)	3824.74 (100%)
Ireland 3	-	-	2514.90 (100%)	-	-	-	-	-	-	2514.90 (100%)
Germany 2	108.48 (26%)	-	-	264.16 (63%)	44.60 (11%)	-	-	-	-	417.24 (100%)
Austria 1	-	145.04 (18%)	-	-	-	-	-	243.60 (30%)	414.26 (52%)	802.90 (100%)
Austria 2	-	145.04 (27%)	-	-	-	-	-	243.60 (45%)	147.63 (28%)	536.27 (100%)
Austria 3	-	-	-	-	-	-	-	121.80 (82%)	26.36 (18%)	148.16 (100%)

Figure 3 Data: Weighted Average Capacity Cost Rate (Percentage of total salary cost (Int $) across personnel type and site)

For peer review only

For peer review only - http://bmjopen.bmj.com/site/about/guidelines.xhtml

Cost Driver 3: Number of Attendees at Each Programme

Programme	Number of participants
UK 1	13
UK 2	10
UK 3	9
Taiwan	1
Israel 1	10
Israel 2	15
Ireland 1	9
Ireland 2	11
Ireland 3	20
Germany 2	8
Austria 1	12
Austria 2	6
Austria 3	7*

Figure 4 Data: Number of Patients who Attend each Programme Course

*This programme runs for insulin and non-insulin users – insulin typically have 4 patient attendees and non-insulin typically have 9 patient attendees. We took the median number = 6.5 rounded to 7.

For peer review only

Programme	Total Cost	Price Variance from Median	Practitioner Hours	Prac Hrs Variance from Median	*Weighted Average CCR*	*Weighted Average CCR* Variance from Median	No. of Patients	No. of Patients Variance from Median
Ire 2	4664	3,898	78	55	3,825	3,022	11	-1
Ire 3	3193	2,427	61	38	2,515	1,712	20	8
UK 1	1697	931	57	34	1,560	757	13	1
Israel 2	1485	719	25	2	482	-321	15	3
UK 2	1248	482	13	-10	452	-351	10	-2
Ire 1	1099	333	19	-4	879	76	9	-3
Aus 1	766	0	23	0	803	0	12	0
Israel 1	633	-133	15	-8	346	-457	10	-2
Aus 2	633	-133	19	-4	536	-267	6	-6
Germany 2	573	-193	19	-4	417	-386	8	-4
UK 3	567	-199	12	-11	369	-434	9	-3
Aus 3	136	-630	5	-18	148	-655	7	-5
Taiwan	14	-752	5	-18	6	-797	1	-11

Median Programme = Austria 1

Figure 5 Data: Variances from the median programme (Austria 1) – variances in practitioner hours, weighted average capacity cost rate and number of patients

For peer review only

Table 1: Diabetes Self-Management Outcomes Framework

Program form	Outcome I. Individual DSM dispositions	Outcome II. AADE7 Behavior	Outcome III. Disease/Health
1. Program Context	4. Increase diabetes specific self-efficacy, focus of control	11. Healthy Eating	
2. Program characteristics	5. Increase diabetes knowledge	12. Being Active	18. Disease related Outcomes
3. Characteristics of program participants (Specifically health literacy)	6. Change attitudes, believes towards diabetes	13. Self-Monitoring	19. Reduced health risks
	7. Raise diabetes awareness	14. Taking Medication	20. Health related quality of life
	8. Increase perceived social support/desirability of DSM behaviors	15. Problem Solving	21. Mental comorbidity
	9. Enhance self-reflection on DSM behaviors	16. Reduced Risk Behavior	
	10. Improve DSM skills	17. Healthy coping	

For peer review only

Note:
The sub-categories of the DSMOF served as a basis for the selection of outcome measures for the study. Items were selected to measure diabetes self-management behaviors, health indicators, health literacy measures and program information as well as socio-demographic information. Thereby all outcome measures are self-reported, i.e. no biomarkers and no HbA1c was measured.

Table 2: Self-Reported Patient Outcomes

	N	Gen Diet	SD	Special Diet	SD	Problem Solving	SD	Reduced Risk Behaviour	SD	Health Coping	SD	Diabetes Health Literacy	SD	Fdhl	SD	CrDhl	SD
UK 1	*2*	0	**0**	1.33	**.94**	0.5	**3.54**	1.25	**0.17**	-1	**2.83**	0.07	**0.2**	0	**0.28**	0.25	**0**
UK2	*21*	0.02	**.73**	-0.21	**1.75**	0.81	**2.89**	0.11	**0.94**	1.33	**3.23**	-0.19	**0.37**	-0.3	**0.48**	-0.17	**0.59**
UK3	*27*	0.09	**1.26**	0.32	**1.01**	1.81	**4**	0.49	**0.84**	0.85	**3.45**	-0.04	**0.35**	-0.09	**0.46**	-0.01	**0.69**
GER2	*5*	0	**1.87**	0.07	**1.44**	2.6	**1.5**	0.89	**1.11**	3.14	**3.58**	0.13	**0.11**	0.11	**0.3**	0.26	**0.5**
IS1	*11*	0.59	**1.11**	-0.39	**2.33**	4.18	**5.96**	0.41	**1.4**	3.73	**3.93**	-0.03	**0.27**	0.07	**0.59**	-0.25	**0.8**
IS2	*64*	0.38	**3**	-0.22	**2.33**	0.58	**5.34**	0.23	**1.75**	1.17	**4.53**	0.07	**0.52**	0.05	**0.91**	0.04	**0.8**
IR1	*13*	0.73	**1.59**	-0.74	**1.42**	5	**4.28**	0.17	**1.71**	3	**4.08**	0.21	**0.59**	-0.12	**0.74**	0.35	**0.85**
IR2	*5*	-0.2	**0.45**	-0.07	**1.83**	4.6	**5.98**	0.55	**1.05**	3	**2.74**	0.2	**0.45**	0.36	**0.26**	-0.05	**0.59**
AUS1	*6*	0.5	**1.1**		**1.48**	1.5	**3.62**	1.38	**0.92**	3.5	**3.08**	0.07	**0.25**	-0.3	**0.37**	0.42	**0.47**
AUS2	*2*	1.5	**1.12**	1.83	**4.95**	-1.5	**4.95**	3	**1.77**	0.5	**4.95**	0.25	**0.25**	-0.2	**0.42**	0.5	**0.28**
AUS3	*5*	0.3	**.72**	0.93	**3.49**	1.2	**3.49**	0.3	**1.2**	4.4	**3.44**	-0.43	**0.42**	-0.28	**0.46**	-0.75	**0.41**

Figure 6 Data: Self-Reported Patient Outcomes (SD=Standard Deviation)

For Figure 6, General Diet was taken as one example of the health outcome data achieved when mapped with cost per programme. To include each health outcome would make Figure 6 too complex and the main finding that health outcomes were similar across all programmes would not be clear to the reader.

Note:

Behavioral outcomes: Behavioral self-management was assessed by selected subscales of the *Summary of Diabetes Self-care Activities* questionnaire (SDSCA). Participants thereby specified how many days in the last week they have followed a general and diabetes specific diet, exercised, checked their blood glucose level, took their prescribed medication and cared for their feet properly. In addition the reversed scores of the *Problem Areas in Diabetes* questionnaire (PAID20) and the *Appraisal of Diabetes Scale* (ADS) were used as indicators to operationalize diabetes specific problem solving and healthy coping respectively.

Covariates: In addition to the behavioral and health outcomes, socio-demographic variables and health literacy were measured as potential covariates likely to influence the effectiveness of DSM interventions. For socio-demographic variables, information on gender, age, years of education, self-perceived social status measured on a ten point scale, and ethnicity measured by own or parental migration experience was collected. Health literacy was assessed using a six item short form of the HLS-EU questionnaire and a diabetes specific health literacy (DHL) scale. The intervention dose in terms of attended sessions was also assessed, but had to be excluded from the analysis, since some respondents had difficulty in providing this information.

For peer review only

Table 3: Overall effectiveness of DSME programs in Aggregate (*Number of participants* = 366)

Indicator	Baseline	Follow-Up	X² or *t*	*P*	Cohen's *d*
		Behavioral outcomes			
Diet (mean ± SD)	4.49 ± 2.18	4.85 ± 1.92	3.57	< .001	0.187
Diabetes specific diet (mean ± SD)	4.26 ± 1.49	4.34 ± 1.42	0.98	.163	
Exercise ≥ 1 d/wk (%)	78	84	7.22	< .001	
Blood glucose monitoring ≥ 6 d/wk (%)	28	32	3.32	.068	
Taking medication 7 d/wk (%)	88	92	5.30	.021	
Problem areas (mean ± SD)	12.63 ± 5.49	13.76 ± 5.44	5.01	< .001	0.262
Foot care (mean ± SD)	4.18 ± 1.53	4.72 ± 1.53	6.73	< .001	0.362
Appraisal of diabetes (mean ± SD)	24.77 ± 4.56	25.83 ± 4.71	5.14	< .001	0.269
		Disease/health outcomes			
BMI (mean ± SD)	30.11 ± 6.60	29.89 ± 6.43	3.45	< .001	0.181
Health related quality of life (mean ± SD)	53.73 ± 19.33	57.90 ± 19.54	4.88	< .001	0.255
Affective well-being (mean ± SD)	59.24 ± 24.82	61.78 ± 23.87	2.21	.014	0.115

Figure 6 Data: Overall effectiveness of DSME programs in Aggregate (Source: Peer et al., 2016)

Note:

Disease/Health outcomes: Three disease and health outcomes were assessed: (1) the BMI was calculated to assess health risks, (2) the general health perception subscale of the *SF36* was used to quantify health related quality of life, and (3) the *WHO-5* was used to operationalize affective well-being as a reverse indicator for mental comorbidity.

For peer review only

CHEERS Checklist
Items to include when reporting economic evaluations of health interventions

The **ISPOR CHEERS Task Force Report**, *Consolidated Health Economic Evaluation Reporting Standards (CHEERS)—Explanation and Elaboration: A Report of the ISPOR Health Economic Evaluations Publication Guidelines Good Reporting Practices Task Force,* provides examples and further discussion of the 24-item CHEERS Checklist and the CHEERS Statement. It may be accessed via the *Value in Health* or via the ISPOR Health Economic Evaluation Publication Guidelines – CHEERS: Good Reporting Practices webpage: http://www.ispor.org/TaskForces/EconomicPubGuidelines.asp

For peer review only

Section/item	Item No	Recommendation	Reported on page No/ line No
**Title and abstract**			
Title	1	Identify the study as an economic evaluation or use more specific terms such as "cost-effectiveness analysis", and describe the interventions compared.	p.4/ 131
Abstract	2	Provide a structured summary of objectives, perspective, setting, methods (including study design and inputs), results (including base case and uncertainty analyses), and conclusions.	p.2/ 50-78
**Introduction**			
Background and objectives	3	Provide an explicit statement of the broader context for the study. Present the study question and its relevance for health policy or practice decisions.	p.4/ 138-142
**Methods**			
Target population and subgroups	4	Describe characteristics of the base case population and subgroups analysed, including why they were chosen.	p.6/ 204-211
Setting and location	5	State relevant aspects of the system(s) in which the decision(s) need(s) to be made.	p.2/ 54-55
Study perspective	6	Describe the perspective of the study and relate this to the costs being evaluated.	p.5-6/ 188-201
Comparators	7	Describe the interventions or strategies being compared and state why they were chosen.	p.6/ 204-211
Time horizon	8	State the time horizon(s) over which costs and consequences are being evaluated and say why appropriate.	p.6/ 211
Discount rate	9	Report the choice of discount rate(s) used for costs and outcomes and say why appropriate.	N/A
Choice of health outcomes	10	Describe what outcomes were used as the measure(s) of benefit in the evaluation and their relevance for the type of analysis performed.	p.5-6/ 194-201
Measurement of effectiveness	11a	*Single study-based estimates:* Describe fully the design features of the single effectiveness study and why the single study was a sufficient source of clinical effectiveness data.	p.6-8/ 213-269

			N/A
	11b	*Synthesis-based estimates:* Describe fully the methods used for identification of included studies and synthesis of clinical effectiveness data.	N/A
Measurement and valuation of preference based outcomes	12	If applicable, describe the population and methods used to elicit preferences for outcomes.	p.5-6/ 194-201
Estimating resources and costs	13a	*Single study-based economic evaluation:* Describe approaches used to estimate resource use associated with the alternative interventions. Describe primary or secondary research methods for valuing each resource item in terms of its unit cost. Describe any adjustments made to approximate to opportunity costs.	p.4-5/ 146-201
	13b	*Model-based economic evaluation:* Describe approaches and data sources used to estimate resource use associated with model health states. Describe primary or secondary research methods for valuing each resource item in terms of its unit cost. Describe any adjustments made to approximate to opportunity costs.	N/A
Currency, price date, and conversion	14	Report the dates of the estimated resource quantities and unit costs. Describe methods for adjusting estimated unit costs to the year of reported costs if necessary. Describe methods for converting costs into a common currency base and the exchange rate.	p.7/ 243-206
Choice of model	15	Describe and give reasons for the specific type of decision-analytical model used. Providing a figure to show model structure is strongly recommended.	N/A
Assumptions	16	Describe all structural or other assumptions underpinning the decision-analytical model.	N/A
Analytical methods	17	Describe all analytical methods supporting the evaluation. This could include methods for dealing with skewed, missing, or censored data; extrapolation methods; methods for pooling data; approaches to validate or make adjustments (such as half cycle corrections) to a model; and methods for handling population heterogeneity and uncertainty.	p.6-8/ 214-269
**Results**			
Study parameters	18	Report the values, ranges, references, and, if used, probability distributions for all parameters. Report reasons or sources for distributions used to represent uncertainty where appropriate. Providing a table to show the input values is strongly recommended.	N/A
Incremental costs and outcomes	19	For each intervention, report mean values for the main categories of estimated costs and outcomes of interest, as well as mean differences between the comparator groups. If applicable, report incremental cost-effectiveness ratios.	p 8-13/ 272-348
Characterising uncertainty	20a	*Single study-based economic evaluation:* Describe the effects of sampling uncertainty for the estimated incremental cost and incremental effectiveness parameters, together with the impact	N/A

For peer review only

		of methodological assumptions (such as discount rate, study perspective).	N/A
	20b	*Model-based economic evaluation:* Describe the effects on the results of uncertainty for all input parameters, and uncertainty related to the structure of the model and assumptions.	N/A
Characterising heterogeneity	21	If applicable, report differences in costs, outcomes, or cost-effectiveness that can be explained by variations between subgroups of patients with different baseline characteristics or other observed variability in effects that are not reducible by more information.	p.8-13/ 272-348
**Discussion**			
Study findings, limitations, generalisability, and current knowledge	22	Summarise key study findings and describe how they support the conclusions reached. Discuss limitations and the generalisability of the findings and how the findings fit with current knowledge.	p.13-17/ 350-495
**Other**			
Source of funding	23	Describe how the study was funded and the role of the funder in the identification, design, conduct, and reporting of the analysis. Describe other non-monetary sources of support.	p.17/ 498-501
Conflicts of interest	24	Describe any potential for conflict of interest of study contributors in accordance with journal policy. In the absence of a journal policy, we recommend authors comply with International Committee of Medical Journal Editors recommendations.	p.18/ 530

For consistency, the CHEERS Statement checklist format is based on the format of the CONSORT statement checklist

The **ISPOR CHEERS Task Force Report** provides examples and further discussion of the 24-ite[illegible] CHEERS Checklist and the CHEERS Statement. It may be accessed via the *Value in Health* link [illegible] ISPOR Health Economic Evaluation Publication Guidelines – CHEERS: Good Reporting Practice[illegible] webpage: http://www.ispor.org/TaskForces/EconomicPubGuidelines.asp

The citation for the CHEERS Task Force Report is:
Husereau D, Drummond M, Petrou S, et al. Consolidated health economic evaluation reporting standards (CHEERS)—Explanation and elaboration: A report of the ISPOR health economic evaluations publication guidelines good reporting practices task force. Value Health 2013;16:231-50.

For peer review only
